# Boosting Oxygen Reduction Reaction Selectivity in Metal Nanoparticles with Polyoxometalates

**DOI:** 10.1002/smtd.202301805

**Published:** 2024-03-22

**Authors:** Eugenia Pilar Quirós‐Díez, Carlos Herreros‐Lucas, José Manuel Vila‐Fungueiriño, Lucía Vizcaíno‐Anaya, Yolanda Sabater‐Algarra, María del Carmen Giménez‐López

**Affiliations:** ^1^ Centro Singular de Investigación en Química Biolóxica e Materiais Moleculares (CiQUS) Departamento de Química Inorgánica Universidade de Santiago de Compostela Santiago de Compostela 15782 Spain; ^2^ Centro Singular de Investigación en Química Biolóxica e Materiais Moleculares (CiQUS) Departamento de Química Física Universidade de Santiago de Compostela Santiago de Compostela 15782 Spain

**Keywords:** carbon nanofibers, covalent functionalization, gold nanoparticles, oxygen reduction reaction, polyoxometalates, selectivity

## Abstract

The lack of selectivity toward the oxygen reduction reaction (ORR) in metal nanoparticles can be linked to the generation of intermediates. This constitutes a crucial constraint on the performance of specific electrochemical devices, such as fuel cells and metal–air batteries. To boost selectivity of metal nanoparticles, a novel methodology that harnesses the unique electrocatalytic properties of polyoxometalates (POM) to scavenge undesired intermediates of the ORR (such as HO_2_^−^) promoting selectivity is proposed. It involves the covalent functionalization of metal nanoparticle's surface with an electrochemically active capping layer containing a new sulfur‐functionalized vanadium‐based POM (AuNP@POM). To demonstrate this approach, preformed thiolate Au(111) nanoparticles with a relatively poor ORR selectivity are chosen. The dispersion of AuNP@POM on the surface of carbon nanofibers (CNF) enhances oxygen diffusion, and therefore the ORR activity. The resulting electrocatalyst (AuNP@POM/CNF) exhibits superior stability against impurities like methanol and a higher pH tolerance range compared to the standard commercial Pt/C. The work demonstrates for the first time, the use of a POM‐based electrochemically active capping layer to switch on the selectivity of poorly selective gold nanoparticles, offering a promising avenue for the preparation of electrocatalyst materials with improved selectivity, performance, and stability for ORR‐based devices.

## Graphical abstract

Enhancing electrocatalyst selectivity in electrochemical devices is crucial. The innovative approach shows that POM clusters can be employed to scavenge undesired ORR intermediates in poorly selective AuNP electrocatalysts, boosting selectivity (AuNP@POM). The resulting electrocatalyst (AuNP@POM/CNF) supported on carbon nanofibers demonstrates superior stability, enhanced ORR activity, and broader pH tolerance than standard Pt/C. A promising step forward for improved ORR‐based devices.

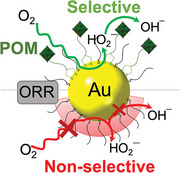

## 1 Introduction

The oxygen reduction reaction (ORR) is a slow electrochemical process that takes place at the cathode of various electrochemical cells, such as fuel cells and metal–air batteries, where it constitutes the main limiting factor.^[^ ^1^, ^2^ ^]^ Improving the activity and efficiency of the ORR in alkaline solutions is a challenging task, largely due to the overproduction of HO_2_^−^ species on the catalyst surface. This issue arises from the presence of a non‐direct 4‐electron (2+2) ORR pathway that involves two separate 2‐electron ORR processes: (i) a 2‐electron HO_2_^−^ formation and (ii) a 2‐electron HO_2_^−^ reduction to OH^−^.^[^ ^3^ ^]^

Current efforts in this area are focused not only on developing active electrocatalyst materials with high selectivity reducing the formation of the HO_2_^−^ intermediate,^[^ ^4^, ^5^, ^6^ ^]^ but also on studying reaction pathways on precious metals to further enhance selectivity.^[^ ^7^, ^8^ ^]^ In this context, intentional surface modification of nanocatalysts with organic ligands has emerged as a promising strategy, not only to control the morphology and crystallographic orientation of nanoparticles, but also to boost selectivity and efficiency in pivotal electrocatalytic processes.^[^ ^7^, ^9^ ^]^ For example, Lagrost et al. demonstrated that the coating of gold nanoparticles (AuNP) with different calix[4]arene derivatives improve the ORR under alkaline conditions compared to their counterparts stabilized with citrate molecules. This study explores the interplay between the length, hydrophobicity, and oxygen affinity of the pendant group in various calix[4]arene derivates. However, no electrochemically active pendant groups were considered.^[^ ^10^ ^]^

On the other hand, polyoxometalates (POM) have shown to be excellent candidates for catalyzing different electrochemical reactions due to their molecular nature, structural diversity, functional tuneability, and high chemical stability.^[^ ^11^, ^12^, ^13^, ^14^ ^]^ As an example, the combination of air‐stable SiW_12_‐ and H_2_W_12_ POM with Pt nanoparticles through electrostatic forces resulted in better ORR activity compared to commercial platinum on carbon black (Pt/C).^[^ ^15^ ^]^ In principle, the use of POM with a swift electrochemical response and sensitivity to reduce HO_2_− could enhance selectivity for the ORR.^[^ ^16^, ^17^, ^18^, ^19^ ^]^ Although the covalent assembly of POM to metallic nanoparticles has already been demonstrated,^[^ ^20^, ^21^, ^22^ ^]^ the applicability of such hybrid materials in electrocatalytic reactions to increase selectivity by reducing undesired intermediates, such as HO_2_^−^, during the ORR, has not been investigated yet.^[^ ^18^ ^]^

To the best of our knowledge, we present, for the first time, a novel methodology for boosting the ORR selectivity in metal nanoparticles. We chose poorly selective Au (111) nanoparticles to demonstrate our approach. It is known that at the Au (111) facet, the ORR takes place via a non‐direct 4‐electron (2+2) pathway, whereas at the low‐indexes Au (100) and Au (110), the ORR exclusively proceeds via a direct one‐step 4‐electron reduction, preventing the formation of hydroperoxyl radicals (HO_2_^−^).^[^ ^8^ ^]^ Our strategy involves the surface modification of octanethiol‐protected Au (111) NP (AuNP@C_8_S) via ligand exchange with an electrochemically active capping layer containing a new sulfur‐functionalized vanadium‐based POM cluster (AuNP@POM) capable of reducing undesired intermediates species, such as the hydroperoxyl radicals (i.e., HO_2_^−^) to increase ORR selectivity. To prevent agglomeration,^[^ ^23^, ^24^, ^25^ ^]^ AuNP@POM is hybridized with carbon nanofibers (AuNP@POM/CNF) increasing ORR activity.^[^ ^26^, ^27^, ^28^, ^29^, ^30^, ^31^ ^]^ The resulting electrocatalyst material (AuNP@POM/CNF) exhibits an enhanced ORR selectivity with respect to AuNP@C_8_S/CNF hybrid material. It also demonstrates superior stability against impurities such as methanol, as well as higher *pH* tolerance compared to the standard commercial Pt/C.

## 2 Results and Discussion

### 2.1 Synthesis and Characterization of a Novel Sulfur‐Functionalized Electroactive POM

**Figure 1:**
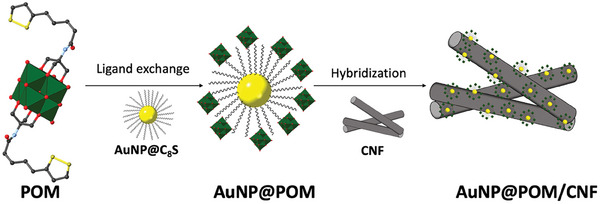
Two‐step preparation scheme for AuNP@POM/CNF. It involves the covalent functionalization of preformed octanethiol gold nanoparticles (AuNP@C_8_S) with POM through a ligand exchange reaction, followed by the hybridization with ball‐milled carbon nanofibers (CNF).^[^ ^32^ ^]^

A novel sulfur‐functionalized hexavanadate cluster [Bu_4_N]_2_[V_6_O_13_{(CH_2_O)_3_CNHCOC_8_H_15_S_2_}_2_] (POM), empirical formula C_56_H_112_N_4_O_21_S_4_V_6_ **, (** **Figure** **1**) was synthesized from the condensation reaction of [Bu_4_N]_3_[H_3_V_10_O_28_] with two lipoic acid derivate molecules ((CH_2_OH)_3_CNHCOC_8_H_15_S_2_). The formation of the hexavanadate cluster from the decavanadate precursor [Bu_4_N]_3_[H_3_V_10_O_28_] was confirmed by mass spectrometry (Figure S4, Supporting Information), while the successful functionalization of the hexavanadate cluster established by thermogravimetric analysis (TGA), infrared (IR) and ^1^H NMR spectroscopy (Figures S5–S7, Supporting Information). Single‐crystal X‐ray diffraction (XRD) of the isolated crystals further corroborates the obtention of a novel POM structure with a monoclinic space group *P*2_1/_ *_n_* composed of anions [V_6_O_13_{(CH_2_O)_3_CNHCOC_8_H_15_S_2_}_2_]^–^ and cations [Bu_4_N]^+^ (Figures S8 and S9; Table S1, Supporting Information). In the crystal structure, POM clusters are connected through hydrogen bonding (between oxygen from the hexavanadate cluster and an amine of the pendant group (O3···H18‐N18)) forming 2D chains along the *a* axis (Figure S10, Supporting Information). The observed V─O bond lengths, ranging from 1.598(3) to 2.2452(6) Å, and the absence of hydrogen atoms bonded to the bridging oxygens (O_b_), confirm the oxidation state of vanadium atoms as V(V) (Table S2, Supporting Information). This is consistent with the observed single‐electron redox process through electrochemical measurements (Figure S11, Supporting Information), which has been also reported for other [V_6_O_13_{(OCH_2_)_3_R}_2_]^2−^ derivates (R = CCH_2_OH, CNH_2_, CNHCOCH_2_Cl).^[^ ^33^ ^]^

### 2.2 Preparation and Structural Characterization of AuNP@POM

**Figure 2:**
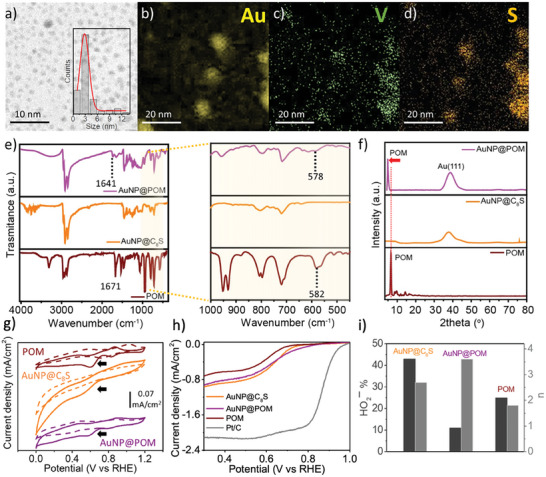
Structural characterization and ORR catalytic behavior of AuNP@POM. a) Bright‐field STEM images at 80 kV (inset: nanoparticle size distribution histogram) and b–d) STEM‐EDS elemental mapping of AuNP@POM. Comparison of FT‐IR e) and powder XRD f) measurements of AuNP@POM with AuNP@C_8_S and POM. g) Comparison of CV measurements in both an oxygen‐ and a nitrogen‐saturated electrolyte (1 m KOH) (black arrows indicates where the ORR occurs), h) LSV measurements at 1600 rpm in an oxygen‐saturated electrolyte, and i) the HO_2_^−^ % (in black) and the number of electrons (*n*) (in grey), obtained for a film of AuNP@POM, AuNP@C_8_S and POM (14 µg cm^−2^) on a glassy carbon electrode.

The sulfur‐functionalized hexavanadate cluster was covalently assembled on the surface of ≈2.5 nm preformed spherical octanethiol gold nanoparticles (AuNP@C_8_S) via a simple ligand exchange reaction involving the partial displacement of octanethiol molecules from the Au surface yielding AuNP@POM (Figure 1). High‐resolution transmission electron microscopy (HR‐TEM) and scanning electron microscopy combined with energy‐dispersive X‐ray spectroscopy (STEM‐EDS) show a homogeneous distribution of vanadium on the surface of the obtained spherical AuNP@POM nanoparticles (**Figure** **2**). No morphological changes were noticed by HR‐TEM after the ligand exchange (Figure 2; Figure S12, Supporting Information). Nevertheless, the size distribution analysis of AuNP@POM shows a slight increase in the average particle size with respect to AuNP@C_8_S, from 2.5 ± 1.3 (Figure S12, Supporting Information) to 2.8 ± 2.2 nm (Figure 2a), respectively, which is in agreement with UV–vis measurements (Figure S13, Supporting Information). However, no changes were observed in the interplanar distances of the gold atoms after the assembly of POM on AuNP@C_8_S with a value of ≈ 2.35 Å that is in agreement with Au(111) (Figure S14, Supporting Information). As expected, TGA measurements of AuNP@POM revealed a higher weight loss at 1000 °C (61%) compared to that observed for AuNP@C_8_S (23%) (Figure S15, Supporting Information).

The successful covalent assembly of POM on the Au surface was confirmed by IR spectroscopy with the presence of the characteristic peaks from the hexavanadate cluster (V─O_t_ (≈ 950 cm^−1^) and V─O_b_ (between 700 and 550 cm^−1^)) (Figure 2), together with the absence of the peak associated to the S─S stretching mode (3314 cm^−1^). The red shift observed after assembly for the IR peaks assigned to POM (i.e., C═O from 1671 to 1641 cm^−1^; V─O_b_ (bridge oxygen), from 582 to 578 cm^−1^) suggests an electron transfer from the Au surface to the POM.^[^ ^34^ ^]^ Furthermore, V─O_t_ (terminal oxygen) bands changed from the appearance of two peaks at 932 and 952 cm^1^ to a broad signal at 954 cm^−1^, confirming the formation of AuNP@POM. Apart from the presence of V atoms, X‐ray photoelectron spectroscopy (XPS) measurements further corroborate such electron transfer based on the increase of the binding energy of S (i.e., from 161.7 to 162.7 eV) and Au (from 87.1 to 87.9 eV) when comparing with AuNP@C_8_S (Figures S16 and S17, Supporting Information).^[^ ^9^ ^]^ Powder XRD measurements of AuNP@POM showed peaks that can be assigned to both AuNP and POM (Figure 2). Thus, a peak at 38.18° for 2θ can be assigned for Au(111) which is also present in AuNP@C_8_S. A shift to lower 2θ values from 6.6 to 4.5° is observed for the peak corresponding to the plane (011) assigned to POM, suggesting that the distance between the hexavanadate clusters when assembled to the Au surface increases compared to that observed in the POM crystal structure. No such change is observed for a physical mixture of POM with preformed octanethiol gold nanoparticles (Figure S18, Supporting Information), highlighting the effect of the covalent assembly of POM on the Au surface.

### 2.3 The Effect of POM on a Gold Surface Toward the ORR

Cyclic voltammogram (CV) measurements conducted on the film materials deposited on a glassy carbon electrode with a loading of 14 µg cm^−2^ in an oxygen‐saturated aqueous electrolyte (1 m KOH) (Figure 2) reveal that AuNP@POM can reduce oxygen molecules, similar to the observed behavior of both POM and AuNP@C_8_S. However, linear sweep voltammetry (LSV) measurements in these films demonstrate a performance enhancement upon the assembly of POM on the surface of AuNP@C_8_S. AuNP@POM exhibits the smallest differences in the onset potential (E_0_) value when compared to the value obtained for the commercial Pt/C standard (Figure 2; Table S3, Supporting Information). Moreover, AuNP@POM shows an enhanced selectivity, as evidenced by lower HO_2_^−^% production (11%) and a number of electrons (n) closer to 4 (3.77) (calculated from the Koutecký–Levich plot), in contrast to that observed for POM (25%, n = 2.56) and AuNP@C_8_S (43%, n = 3.14) (Figure S19, Supporting Information). To verify that the enhanced selectivity observed in AuNP@POM is indeed attributed to POM, we investigated the electrocatalytic behavior of POM toward peroxide (HO_2_^−^) reduction in a basic medium (1 m KOH) and compared it with AuNP@C_8_S (Figure S20, Supporting Information). CV measurements revealed only for POM a significant increase in current density with increasing peroxide (HO_2_^−^) concentrations.

### 2.4 Structural and Electrochemical Characterization of AuNP@POM/CNF

**Figure 3:**
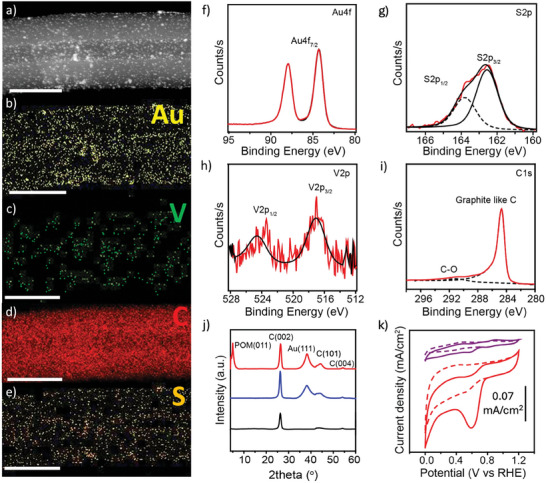
Structural and electrochemical characterization of AuNP@POM/CNF. a) Dark‐field HR‐TEM images at 80 kV using a copper grid. STEM‐EDS elemental mapping for AuNP@POM/CNF showing Au b), V c), C d), and S e). The scale bars are 100 nm. XPS measurements for the energy regions of f) Au4f, g) S2p, h) V2p, and i) C1s. j) Comparison of powder XRD measurements of AuNP@POM/CNF (red) with AuNP@C_8_S/CNF (blue) and CNF (black). k) Comparison of cyclic voltammograms for films of AuNP@POM/CNF and AuNP@POM deposited onto a glassy carbon electrode (14 µg cm^−2^ loading) in both an oxygen‐ and nitrogen‐saturated electrolyte (1 m KOH).

AuNP@POM was hybridized with ball‐milled carbon nanofibers (CNF, Figure S4, Supporting Information) by simply mixing in hexane at a ratio of 2.5:1 (wt.), respectively (Figure 1). After annealing at 800 °C, the ashes estimated by TGA for AuNP@POM/CNF were higher (17.2%) than those found for the AuNP@POM/gCNF hybrid material with graphitized carbon nanofibers (gCNF) (15.6%) (Figure S21, Supporting Information). It is proposed that oxygen‐containing groups present on the surface of the carbon nanofibers, as a consequence of the ball‐milling treatment (I_D_/I_G_ of 0.30 and C─O component in Figure S3, Supporting Information), act as anchoring points for AuNP@POM. ICP‐OES analysis of the digested samples revealed Au contents in wt.% of 22.2 ± 0.2 and 21.1 ± 0.1 for AuNP@POM/CNF and AuNP@POM/gCNF, respectively. HR‐TEM measurements reveal a homogeneous distribution of AuNP@POM on the CNF surface (**Figure** **3**; Figure S22, Supporting Information) with an average nanoparticle size of 3.6 ± 2.6 nm, slightly higher than the value found before hybridization with CNF. From IR measurements, it is difficult to undoubtedly ascertain the presence of POM in AuNP@POM/CNF due to overlap with the CNF bands (Figure S23, Supporting Information). However, both XPS and XRD measurements allow for the clear identification of the presence of AuNP@POM in the obtained hybrid material.^[^ ^35^ ^]^ In addition to the presence of Au, S, and V atoms by XPS (Figure 3; Figure S24, Supporting Information), powder XRD measurements of AuNP@POM/CNF showed peaks that can be assigned to POM at low 2θ values, as well as the existence of Au(111) peaks also present in AuNP@C_8_S/CNF (Figure 3 and Figure S25, Supporting Information). Therefore, the surface composition of AuNP@POM after hybridization with CNF remains unchanged.

The cyclic voltammogram of a film of AuNP@POM/CNF deposited on a glassy carbon electrode with a loading of 14 µg cm^−2^ (0.1 m TBAPF_6_ in acetonitrile) exhibits a one‐electron redox process associated with the V^V^/V^IV^ couple,^[^ ^33^ ^]^ featuring a formal potential of −0.545 V (vs Ferrocene) and a peak separation of 144 mV. However, this process occurs 214 mV higher than the formal potential observed for AuNP@POM with a peak separation of 203 mV, suggesting that after hybridization, CNF drains electron density from the AuNP@POM and improves conductivity (Figure S26, Supporting Information). It is suggested that this electron transfer may enhance the electrocatalytic behavior of AuNP@POM/CNF toward the ORR.^[^ ^36^ ^]^ A comparison of the cyclic voltammograms conducted for AuNP@POM/CNF and AuNP@POM deposited on a glassy carbon electrode reveals a sharp, irreversible reduction peak around 0.6 V in the presence of an oxygen‐saturated aqueous electrolyte (1 m KOH), demonstrating the enhanced reduction capability of AuNP@POM after hybridization.

### 2.5 Catalytic Activity and Selectivity of AuNP@POM/CNF Toward the ORR

**Figure 4:**
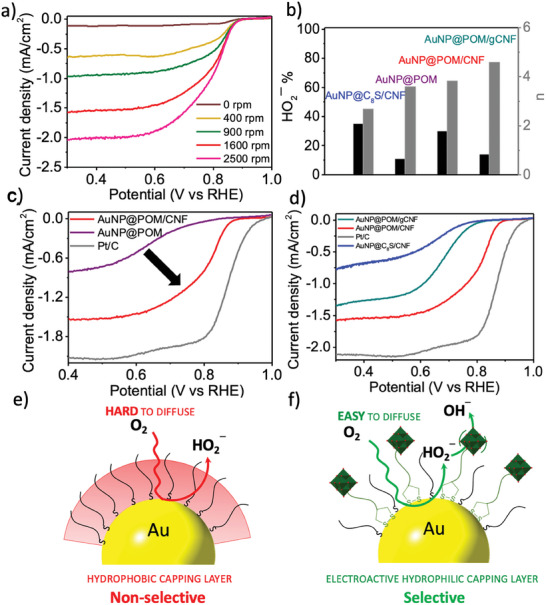
Activity and selectivity of a film of AuNP@POM/CNF deposited onto a glassy carbon electrode toward the ORR. a) LSV for a film of AuNP@POM/CNF at different rotating speeds (0‐2500 rpm) in an oxygen‐saturated 1 m KOH solution. b) HO_2_^−^ % (in black) and the number of electrons (*n*) (in grey) involved in the ORR at 1600 rpm in an oxygen‐saturated 1 m KOH solution for AuNP@C_8_S, AuNP@POM, AuNP@POM/CNF and AuNP@POM/gCNF. Comparison of LSV at 1600 rpm in an oxygen‐saturated 1 m KOH solution for films of c) AuNP@POM and d) AuNP@C_8_S, and AuNP@POM/gCNF with AuNP@POM/CNF and the Pt/C standard deposited onto a glassy carbon electrode under the same conditions (14 µg cm^−2^). The selectivity behavior proposed toward the ORR for e) AuNP@C_8_S with a hydrophobic capping layer and for f) AuNP@POM with an electroactive, hydrophilic capping layer.

After hybridizing AuNP@POM with CNF, an important increase in the diffusion‐limiting current density (j_L_) (from −0.90 to −1.60 mA cm^−2^ at 0.4 V at 1600 rpm), as well as in the onset (from 753 to 868 mV) and the half‐wave potential (from 660 to 790 mV) values is observed for AuNP@POM/CNF as shown by LSV measurements under alkaline conditions (1 m KOH) (**Figure** **4**; Table S3, Supporting Information). The values for the Tafel slope (−47.4 mV dec^−1^) obtained and the number of electrons transferred (*n* = 3.85) calculated from the *K–L* plot (Figure S27, Supporting Information) suggest that AuNP@POM/CNF can easily adsorb and facilitate the reduction of oxygen molecules through a non‐direct 4‐electron pathway.^[^ ^37^ ^]^ It is suggested that an enhancement of the hydrophilic character of the capping layer, attributed to the presence of POM, may facilitate the oxygen diffusion to the gold surface when AuNP@POM nanoparticles are effectively dispersed on a conductive support, thereby enhancing activity (Figure 4).^[^ ^38^ ^]^ In fact, when AuNP@C_8_S nanoparticles that are much more hydrophobic than AuNP@POM (Figure S28, Supporting Information) are supported on CNF, no significant enhancement is noted as the diffusion‐limiting current density (j_L_) and half‐wave potential values remain nearly unchanged (Figure S29, Supporting Information).

The ring current density for AuNP@POM/CNF, which is used to identify undesired intermediates such as HO_2_^−^, reveals the production of a higher amount of this species (30%) compared to that observed for AuNP@POM (11%) (Figure 4). It has been reported that the presence of oxygen‐containing groups on carbon supports is involved in the production of HO_2_^−^.^[^ ^39^ ^]^ In fact, the electrochemical characterization of CNF shows the generation of a high percentage of HO_2_^−^ intermediate (75%) via 2‐electron pathway (*n* ≈ 2.07) (Figure S29, Supporting Information). When graphitized carbon nanofibers (gCNF) with a reduced number of oxygen defects are used as support for AuNP@POM, a reduction in the production of HO_2_^−^ is observed (14%) favoring the selectivity of the ORR toward the formation of OH^−^ (Figure S30, Supporting Information). However, this increase in selectivity accompanies an important decrease in activity due to an increase in the hydrophobic character of the carbon support, showing lower values of j_L_ (−1.34 mA cm^−2^) and half‐wave potential (680 mV) (Figure 4; Table S3, Supporting Information).

### 2.6 ORR Stability and Methanol Tolerance of AuNP@POM/CNF

**Figure 5:**
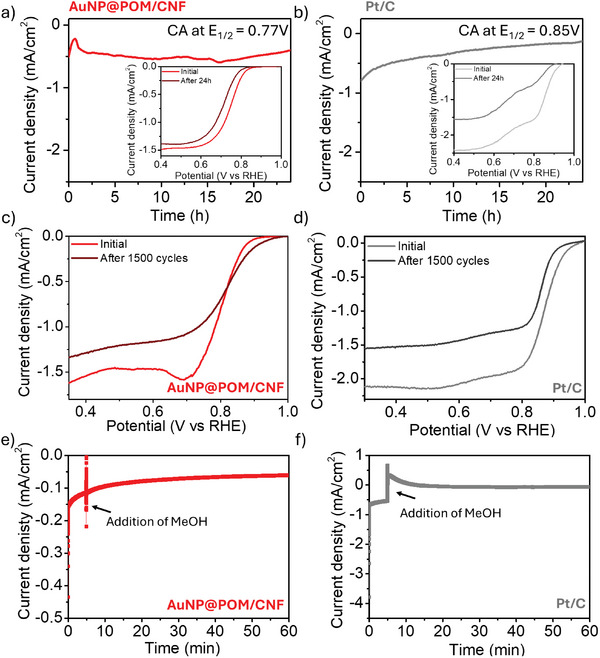
24‐h chronoamperometry (CA) measurements with the potential held at E_1/2_ = 0.77 V and E_1/2_ = 0.85 V for a film of AuNP@POM/CNF a) and Pt/C b) deposited on a glassy carbon electrode (14 µg cm^−2^), respectively (inset: polarization curves at 1600 rpm at different times). Polarization curves at 1600 rpm before and after 1500 cycles in an oxygen‐saturated solution of 1 m KOH for AuNP@POM/CNF c) and Pt/C d). Methanol tolerance evaluation for a film of AuNP@POM/CNF (e) and Pt/C f) deposited on a glassy carbon electrode (14 µg cm^−2^) with the addition of 4 mL methanol after 300 s chronoamperometry measurements are initiated with the potential set to E_1/2_ = 0.76 V and E_1/2_ = 0.84 V, respectively.

To assess the electrochemical stability of a film of AuNP@POM/CNF deposited on a glassy carbon electrode, accelerated durability tests were carried out using both chronoamperometry and cyclic voltammetry techniques. Changes in the ORR activity of the hybrid catalyst were evaluated through LSV measurements before and after durability tests. After chronoamperometry measurements (potentially held at E_1/2_ for 24 h), minor changes in terms of onset potential, as well as a small shift of 25 mV in the half‐wave potential value were observed for AuNP@POM/CNF. This material exhibited ≈ 80% retention of the current density value. As shown in **Figure** **5**, this result confirms the suitability of our material compared to standard Pt/C, which retains only 16% of its current under the same conditions. In Table S4 (Supporting Information) the observed retention values are compared with other electrocatalysts reported in the literature in the last 2 years. After performing 1500 repetitive cyclic voltammetry scans for the ORR, a smaller current density decay for AuNP@POM/CNF (20%) compared to Pt/C standard under the same conditions (30%) is observed (Figure 5).

In addition, AuNP@POM/CNF deposited onto a glassy carbon electrode exhibits a good *pH* and methanol tolerance. For instance, the limiting current observed for AuNP@POM/CNF decreases by only 37% when changing from a 0.1 to 1 m KOH solution, without affecting the electrochemical pathway for the ORR (Figure S31, Supporting Information), whereas the Pt/C standard experiences a 52% decrease in the limiting current density with the same change in *pH* (Figure S32, Supporting Information). Methanol tolerance was assessed by adding 4 mL of methanol after 300 s chronoamperometry measurements were initiated with the potential set to E_1/2_. AuNP@POM/CNF exhibited minimal fluctuation in the current output, in contrast to that observed for the Pt/C standard (Figure 5).

## 3 Conclusion

We demonstrated for the first time a new methodology for the surface modification of gold nanoparticles (AuNP@C_8_S) with an electroactive capping layer (POM) allowing the selectivity increase of a non‐direct 4‐electron ORR process in a basic medium. We rationally designed an electroactive sulfur‐functionalized vanadium‐based POM cluster (POM) that has proven to be highly active toward hydrogen peroxide reduction. This new POM can be covalently assembled on the surface of gold metal nanoparticles through Au─S bonds that involve an electron transfer from the Au to the POM. The electroactive capping layer in AuNP@POM reacts with undesired intermediates (HO_2_^−^), boosting the selectivity of a non‐direct 4‐electron ORR process, as demonstrated by LSV measurements with a Rotating Ring‐Disk Electrode. In addition, an important increase in the ORR activity is observed upon hybridization CNF that allows the effective dispersion of AuNP@POM on a conductive carbon support. The AuNP@POM/CNF hybrid combines good stability and superior methanol and *pH* tolerance compared to the Pt/C standard. The more hydrophilic character of the surfactant layer in AuNP@POM compared to AuNP@C_8_S enhances the oxygen diffusion to the gold surface, and therefore its ORR activity, whereas the absence of oxygen‐defects in the carbon support reduces the HO_2_^−^ production favoring the ORR selectivity toward the OH^−^. This new strategy developed in this work can be extended to other metal surfaces and POM clusters with different activities, producing a new generation of energy‐sustainable materials.

## 4 Experimental Section

### Material

All the chemicals were purchased from Sigma–Aldrich and used without any purification. Carbon nanofibers were purchased from Pyrograf Products Inc., USA, and were first shortened by mechanical ball milling (CNF) and later graphitized (gCNF) under Ar atmosphere, as described in the Supplementary Information (SI). Platinum, nominally 20% on carbon black (Pt/C), HISPEC 3000 was purchased from Alfa Aesar. (CH_2_OH)_3_CNHCOC_8_H_15_S_2_, [Bu_4_N]_3_[H_3_V_10_O_28_] and AuNP@C_8_S were prepared following reported experimental procedures.^[^ ^40^, ^41^, ^42^ ^]^

### Synthesis of [Bu_4_N]_2_[V_6_O_13_{(CH_2_O)_3_CNHCOC_8_H_15_S_2_}_2_] (POM)

In a two‐neck round‐bottom flask, 0.2 g of (CH_2_OH)_3_CNHCOC_8_H_15_S_2_ (0.11 mmol, 1 equiv.) (Figure S1, Supporting Information), 0.11 g of [Bu_4_N]_3_[H_3_V_10_O_28_] (0.32 mmol, 3 equiv.) (Figure S2, Supporting Information) and 4 mL of dry dimethylacetamide were added. The mixture was heated to 85 °C and stirred for 24 h under oxygen before cooling down to room temperature. Subsequently, 10 mL of diethyl ether was added. The resulting solid was decanted and vigorously stirred in 8 mL of acetone for 1 h. The solid, recovered by filtration, was then washed with acetone. Orange needle‐like crystals (POM) (37% of yield) were obtained after recrystallization of the orange powder, using a mixture 1:1:4 of MeCN:DMF:Et_2_O. [Bu_4_N]_2_[V_6_O_13_{(CH_2_O)_3_CNHCOC_8_H_15_S_2_}_2_] (POM), empirical formula C_56_H_112_N_4_O_21_S_4_V_6_. IR (ATR, cm^−1^): 3314 (m); 2959 (s); 2930 (s); 2871 (s); 1971 (s); 1529 (m); 1471 (m); 1069 (m); 1040 (m); 952 (vs); 932 (vs); 796 (m); 720 (s); 581 (m). ^1^H NMR (300 MHz; DMSO; δ (ppm)): 0.94 (t, J = 7.3 Hz, 24H); 1.32 (q, J = 7.3 Hz, 16H); 1.4–1.5 (m, 6H); 1.53–1.61 (m, 18H); 1.61–1.68 (m, 2H); 1.81–1.9 (m, 2H); 2.06 (t, J = 7.1 Hz, 4H); 2.35–2.43 (m, 2H); 3.06–3.12 (m, 2H); 3.15–3.21 (m, 16H); 3.56–3.64 (m, 2H); 5.10 (s, 12H); 7.35 (s, 2H). (Figures S4–S7, Supporting Information)

### Synthesis of AuNP@POM

10 mg of AuNP@C_8_S suspended in 5 mL of dry dichloromethane were added dropwise to a solution of 40 mg of POM in 15 mL of dry dichloromethane. The resulting mixture was vigorously stirred for 4 days under an Ar atmosphere before adding 20 mL of methanol. The residue was isolated by centrifugation at 8000 rpm for 10 min, washed with methanol (6×20 mL), and dried under vacuum to obtain 15 mg of AuNP@POM material.

### Preparation of AuNP@POM/CNF, AuNP@POM/gCNF and AuNP@C_8_S/CNF Hybrids

16.4 mg of carbon nanofibers (either CNF or gCNF) (Figure S3, Supporting Information) suspended in 1 mL of dry hexane were added dropwise to a suspension of 41 mg of either AuNP@POM or AuNP@C_8_S in 4 mL of dry hexane. The resulting mixture was vigorously stirred for 4 days under an Ar atmosphere and then dried by evaporating the solvent at room temperature under reduced pressure. The resulting solid was washed with hexane (3 × 20 mL) and isolated by centrifugation at 8000 rpm for 10 min. The resulting black powder was dried under vacuum to yield either 22 mg of AuNP@POM/CNF or 18.5 mg of AuNP@POM/gCNF or 19.5 mg of AuNP@C_8_S/CNF with a final residue after annealing at 800 °C of 17.2%, 15.6% and 15.0%, respectively.

### Electrochemical Technique

Electrochemical measurements were conducted in both aqueous and organic electrolytes using a standard three‐electrode setup with an AUTOLAB 302N and a Gamry 600+ potentiostat/galvanostat. A glassy carbon rotating ring‐disk electrode (RRDE) was used as the working electrode in aqueous media, and a static glassy carbon electrode was employed for experiments in organic media. A carbon rod and platinum wire served as the counter electrode in aqueous and organic media, respectively, and a reversible hydrogen electrode (RHE, Gaskatel GmbH) was utilized as the reference electrode in aqueous media, while Ag/AgNO_3_ was employed in organic media. Details on ink preparation and modification of the glassy carbon electrode to achieve a final catalyst loading of 14 µg cm^−2^ for all the studied materials are available in the supporting information. Cyclic voltammograms (CV) were conducted in organic media to detect V^V^/V^IV^ electron processes and assess the existence of electron transfer after surface functionalization.^[^ ^33^ ^]^ ORR activity was evaluated through CV and linear sweep voltammograms (LSV) in oxygen‐saturated alkaline aqueous electrolytes (0.1 and 1 m KOH). Catalyst selectivity was assessed through rotating ring‐disk electrode (RRDE) measurements in the 400–2500 rpm rotation range. Supporting information details calculations for electron transfer numbers from the Koutecký–Levich (*K–L*) equations and HO_2_^–^ % yields. Catalyst activity toward H_2_O_2_ reduction was determined via CV in 1 m KOH with varying H_2_O_2_ concentrations. Chronoamperometry (CA) measurements on a rotating disk electrode were employed to evaluate catalyst stability, while methanol tolerance was assessed by introducing a known quantity of methanol during CA measurements.

[CCDC 2252000 contains the supplementary crystallographic data for this paper. These data can be obtained free of charge from The Cambridge Crystallographic Data Centre via www.ccdc.cam.ac.uk/data_request/cif.]

## Conflict of Interest

The authors declare no conflict of interest.

## Supporting information

Supporting Information

## Data Availability

The data that support the findings of this study are available in the supplementary material of this article.
