# Supplementary material for: Boosting Oxygen Reduction Reaction Selectivity in Metal Nanoparticles with Polyoxometalates
Source: Small Methods. 2024 Mar 22;8(12):2301805. doi: 10.1002/smtd.202301805 (PMC11672175; doi:10.1002/smtd.202301805)
Supplement: Supplementary file 1 — Supporting Information [file SMTD-8-2301805-s001.pdf]

small methods

Supporting Information

for *Small Methods*, DOI 10.1002/smtd.202301805

Boosting Oxygen Reduction Reaction Selectivity in Metal Nanoparticles with Polyoxometalates

*Eugenia Pilar Quirós-Díez, Carlos Herreros-Lucas, José Manuel Vila-Fungueiriño, Lucía Vizcaíno-Anaya, Yolanda Sabater-Algarra and María del Carmen Giménez-López**

Supporting Information

Boosting Oxygen Reduction Reaction Selectivity in Metal Nanoparticles with Polyoxometalates

*Eugenia Pilar Quirós-Díez, Carlos Herreros-Lucas, José Manuel Vila-Fungueiriño, Lucía Vizcaíno-Anaya, Yolanda Sabater-Algarra and María del Carmen Giménez-López**

*Corresponding Author: María del Carmen Giménez-López. E-mail address: maria.gimenez.lopez@usc.es

Author Contributions

E.P.Q.-D. and C.H.-L. prepared and characterized all the materials and analyzed the data. J.M.V.-F. performed HR-TEM and STEM/EDS analysis. L.V.-A. contributed to the electrochemical discussion and the 24h-CA measurements. Y.S.-A. contributed to ICP-OES and zeta-potential analysis. M.C.G.-L. contributed to the idea and follow-up of the work, analyzed the data and wrote the final version of the manuscript with contributions of all authors. All authors have given approval to the final version of the manuscript.

Table of content

Electrochemical measurements 2

- **A. Cyclic voltammetry in organic media:** 2
- **B. Ink preparation and glassy carbon electrode modification:** 2
- **C. ORR performance evaluation:** 2
- **D. Rotating Ring-Disk Electrode (RRDE) experiment:** 3
- **E. Stability measurements:** 3
- **G. Activity towards H_2O_2 reduction:** 4

Structural characterization 4

Synthesis and characterization of materials 6

- **H. Synthesis of $(CH_2OH)_3CNHCOC_8H_{15}S_2$:** 6
- **I. Synthesis of $[Bu_4N]_3[H_3V_{10}O_{28}]$:** 6
- **J. Shortening of pristine carbon nanofibers (CNF):** 7
- **K. Graphitization of CNF (gCNF):** 7

Supplementary Figures 8

Electrochemical measurements

A. Cyclic voltammetry in organic media:

CV experiments were conducted in an acetonitrile solution saturated with Ar, incorporating $TBAPF_6$ (0.1 M) as the supporting electrolyte. All potentials were recorded at room temperature relative to $Ag/AgNO_3$. To provide a consistent reference, ferrocene (1 mM) was introduced to the solution at the conclusion of each experiment, serving as an internal standard. Consequently, all electrochemical potentials were normalized to its redox couple (Fc/Fc^+). The diffusion coefficient was calculated using Randles-Sevcik equation (1):

$$I_p = 2.69 \times 10^5 A \times D^{1/2} n^{3/2} v^{1/2} C \quad (1)$$

Where I_p is the anodic peak current, A the area of the electrode (cm^2), D the diffusion coefficient of the electroactive specie (cm^2/s), n the electrons involved in the redox reaction, v the scan rate (V/s) and C the concentration (mol/cm^3).

B. Ink preparation and glassy carbon electrode modification:

Prior to use, glassy carbon (GC) electrodes underwent mechanical polishing with aqueous alumina powder slurries (0.05 µm), followed by rinsing with Milli-Q water and acetone, and drying under nitrogen. For electrode preparation, the catalyst was dispersed in hexane to achieve a concentration of 6.25 mg/mL. After 15 minutes of sonication to create a uniform catalyst ink, it was deposited onto the GC disk (5 mm diameter) to achieve a final catalyst loading of 14 $\mu g/cm^2$. Upon solvent evaporation, the deposited catalyst was coated with a 20 µL drop of a dilute aqueous Nafion solution (0.1 wt%). The resulting thin film proved robust enough to permanently attach the catalyst to the GC surface without introducing any resistance. The prepared electrode was air-dried at room temperature for 20 minutes prior to electrochemical testing.

C. ORR performance evaluation:

The number of electrons transferred (*n*) during the ORR was calculated for all the materials from the rotating disk electrode (RDE) voltametric results obtained at electrode rotation speeds using the Koutecký-Levich equation (2):

$$\frac{1}{J} = \frac{1}{J_L} + \frac{1}{J_K} = \frac{1}{B\omega^{0.5}} + \frac{1}{J_K} \quad (2)$$

$$B = 0.62nFC_o(D_o)^{2/3}\nu^{-1/6} \quad (3)$$

Where J is the measured current density and ω is the electrode rotating rate (rad s^{-1}). The Levich constant, B is determined from the slope of the Koutecký-Levich (K-L) plot based on Levich equation (3). J_L and J_K are the diffusion- and kinetic-limiting current densities, n is the transferred electron number, F is the Faraday constant (F = 96,485 C mol^{-1}), C_0 is the O_2 concentration in the electrolyte ($C_0 = 1.26 \times 10^{-6}$ mol cm^{-3}), D_0 is the diffusion coefficient of O_2 ($D_0 = 1.93 \times 10^{-5}$ cm^2 s^{-1}), and ν is the kinetic viscosity (ν = 0.01009 cm^2 s^{-1}). The constant 0.62 is adopted when the rotation speed is expressed in rad s^{-1}.

D. Rotating Ring-Disk Electrode (RRDE) experiment:

All Rotating Ring-Disk Electrode (RRDE) measurements were conducted in a O_2 saturated KOH (0.1 and 1 M) solution with a ring potential set to 1 V vs. RHE. The four-electron selectivity of catalysts was evaluated based on the H_2O_2 yield and transfer number (n), which was calculated from the following equations (4-5):

$$HO_2^-\% = 200 \times \frac{I_R/N}{I_D + I_R/N} \quad (4)$$

$$n = 4 \times \frac{I_D}{I_D + I_R/N} \quad (5)$$

where I_D is disk current, I_R is ring current, and N is current collection efficiency of the Pt ring (40%).

E. Stability measurements:

The stability of the catalysts was assessed through chronoamperometry (CA) measurements, where the potential was held at $E_{1/2}$ for 1 hour while rotating at 1600 rpm in an O_2-saturated solution of 1 M KOH.

F. Evaluation of methanol tolerance:

The methanol tolerance of the catalysts was evaluated by adding 4 mL of MeOH during CA measurements (i.e., at 300s) with the potential set to $E_{1/2}$ for 1 hour while rotating at 1600 rpm in an O_2-saturated solution of 1 M KOH.

G. Activity towards H_2O_2 reduction:

The electrochemical reduction of H_2O_2 was evaluated by CV (from -0.45 to 0.8 V) of a modified glassy carbon electrode (i.e., 5 mm diameter) in a 1 M KOH solution with different H_2O_2 concentrations (i.e., 2, 10, 20, and 40 mM).

Structural characterization

Infrared spectra were measured using a Bruker Alpha FTIR spectrometer with a platinum ATR module. UV-Vis spectra (190-900 nm) were measured using a V-750 JASCO spectrophotometer. Thermogravimetric analysis (TGA) was performed on a TA Instruments TGA-SDTQ500 analyser. Scanning electron microscopy (SEM) and energy-dispersive X-ray spectroscopy (EDX) analysis were performed on a ZEISS EVO LS 15 with EDX module (Oxford Inca x-act with 129 eV of resolution and WD 8.5 mm). High-resolution transmission electron microscopy (HR-TEM) was performed on a JEOL JEM F200 microscope equipped with a cold field-emission gun (Cold-FEG) operated at 200 kV with an ultra-high-resolution pole piece. TEM images were acquired using a Gatan OneView camera. Energy Dispersive X-ray Spectroscopy (EDX) was performed with a Centurio Large Angle Silicon Drift Detector (SDD) that collects X-rays from a detection area of 100 mm^2. Copper grids were used for HR-TEM measurements. Raman spectra were performed with RENISHAW Raman microscope with laser Ion Ar (514 nm). Single-crystal X-ray diffraction was measured on a Bruker D8 Venture Photon 100 CMOS κ--geometry diffractometer system equipped with an Incoatec high brillance IμS microsource (MoKα, λ = 0.71073 Å) and an Incoatec HeliosTM multilayer optics monochromator. Ball-milling was carried out using a high-energy Retsch MM400 ball mill instrument. X-ray photoelectron spectroscopy (XPS) analysis was performed in a ESCALAB250Xi (ThermoFisherScientific) with a monochromated Al K_α ($h\nu$ = 1486.68eV) radiation, operated at 220 W, 14.6 kV, spot size 650 μm. XPS spectra were collected at 100 eV and 40 eV for survey spectra and individual elements, respectively. The energy step for individual elements was 0.1 eV. The XPS spectra were peak fitted using Advantage data processing software. For peak fitting the Shirley-type background subtraction was used. All the XPS peaks are to be referenced to adventitious carbon C1s, C-C peak at 284.8 eV. Quantification has been done using sensitivity factors provided by the Avantage library. Charge neutralization was achieved with both low energy electron and argon ion Flood guns (0.5e, 100 μA and 25 μA current respectively) during XPS measurements. NMR analysis was performed in a Varian Mercury 300 MHz

spectrometry. Mass spectrometry analysis was performed in a Bruker Microtof. X-Ray Diffraction was measured on a PaNalytical-EMPYREAM equipped with a theta/theta-two goniometer using Cu anode with a PIXCel3D type hybrid solid state detector. The Au content for AuNP@POM/CNF and AuNP@POM/gCNF was determined by ICP-OES using a PerkinElmer Optima 3300 DV ICP-OES. The catalyst samples were treated with a mixture of acids (69% HNO_3 and 35% HCl). The residues were transferred to Teflon vessels and introduced into a laboratory digester (Easy Ethos Milestone). Zeta (ζ) potentials of AuNP@C_8S and AuNP@POM re-dispersed in miliQ water were measured using a Zetasizer Nano ZS (Malvern Instrument Ktd.) by using a disposable capillary zeta cell. ζ potential measurements reported are an average of five independent measurements.

Single-crystal X-ray diffraction measurements for POM ($[Bu_4N]_2[V_6O_{13}\{(CH_2O)_3CNHCOC_8H_{15}S_2\}_2]$)

Orange needle-like specimen of $C_{56}H_{112}N_4O_{21}S_4V_6$, with approximate dimensions 0.027 mm × 0.042 mm × 0.159 mm, was used for the X-ray crystallographic analysis. The X-ray intensity data were measured on a Bruker D8 Venture Photon 100 CMOS κ--geometry diffractometer system equipped with an Incoatec high brillance IµS microsource (MoKα, λ = 0.71073 Å) and an Incoatec HeliosTM multilayer optics monochromator. A total of 1269 frames were collected. The total exposure time was 27.73 hours. The frames were integrated with the Bruker SAINT software package using a narrow-frame algorithm. The integration of the data using a monoclinic unit cell yielded a total of 68214 reflections to a maximum θ angle of 28.28° (0.75 Å resolution), of which 9677 were independent (average redundancy 7.049, completeness = 99.8%, R_{int}=11.59%, R_{sig}=8.50%) and 6192 (63.99%) were greater than 2σ(F^2). The final cell constants of a = 10.6356(5) Å, b = 19.0646(8) Å, c = 19.9100(9) Å, β = 104.612(2)°, volume = 3906.4(3)$Å^3$, are based upon the refinement of the XYZ-centroids of 9927 reflections above 20 σ(I) with 4.737° < 2θ < 56.86°. Data was corrected for absorption effects using the Multi-Scan method (SADABS). The ratio of minimum to maximum apparent transmission was 0.824. The calculated minimum and maximum transmission coefficients (based on crystal size) are 0.8750 and 0.9770. The structure was solved and refined using the Bruker SHELXTL Software Package, using the space group $P2_1/n$, with Z = 2 for the formula unit, $C_{56}H_{112}N_4O_{21}S_4V_6$. The final anisotropic full-matrix least-squares refinement on F^2 with

661 variables converged at R1 = 6.55%, for the observed data and wR2 = 14.68% for all data. The goodness-of-fit was 1.027. The largest peak in the final difference electron density synthesis was 0.678 $e^{-}/Å^3$ and the largest hole was -0.630 $e^{-}/Å^3$ with an RMS deviation of 0.091 $e^{-}/Å^3$. On the basis of the final model, the calculated density was 1.370 g/cm^3 and F(000) = 1692 e^{-}.

Synthesis and characterization of materials

H. Synthesis of $(CH_2OH)_3CNHCOC_8H_{15}S_2$:

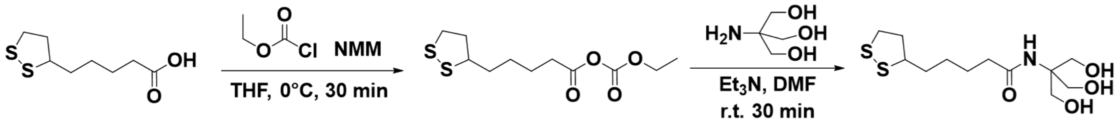

A previous reported method was followed [1].

^{1}H NMR (300 MHz, Methanol-*d*$_4$) δ 3.71 (s, 6H), 3.58 (dq, *J* = 8.8, 6.4 Hz, 1H), 3.23 – 3.02 (m, 2H), 2.56 – 2.37 (m, 1H), 2.26 (t, *J* = 7.4 Hz, 2H), 2.00 – 1.82 (m, 1H), 1.76 – 1.56 (m, 4H), 1.57 – 1.32 (m, 2H)

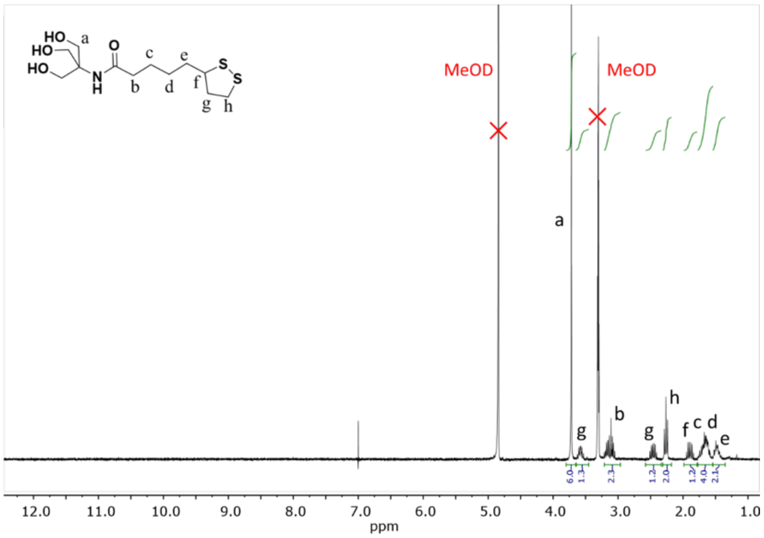

Figure S1. ^{1}H NMR measurements in Methanol-*d*$_4$ at 300 MHz of the ligand $[(CH_2OH)_3CNHCOC_8H_{15}S_2]$.

I. Synthesis of $[Bu_4N]_3[H_3V_{10}O_{28}]$:

$$10Na_3VO_4 + 27HCl_{(aq)} + 3Bu_4NBr \longrightarrow [Bu_4N]_3[H_3V_{10}O_{28}] + 3NaBr + 27NaCl + 12H_2O$$

A previous reported method was followed [2].

$[Bu_4N]_3[H_3V_{10}O_{28}]$. IR (ATR, cm^{-1}): 2959 (s); 2931 (m); 2870 (s); 1481 (s); 1378 (s); 983 (m); 967 (s); 944 (m); 882 (m); 837 (m); 801 (m); 763 (m). SCXRD (100 K): a: 17.3738(11) Å, b: 27.7778(18) Å, c: 32.528(2) Å.

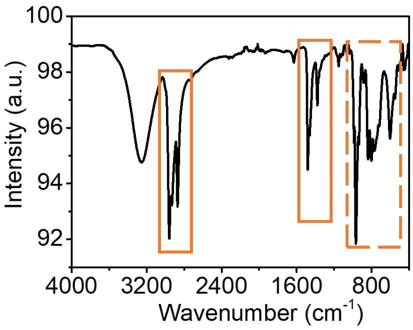

Figure S2. FT-IR measurements of **$[Bu_4N]_3[H_3V_{10}O_{28}]$**.

J. Shortening of pristine carbon nanofibers (CNF):

As received carbon nanofibers were shortened by mechanical ball milling to yield CNF. In a typical experiment, 50 mg of as received carbon nanofibers were placed into a stainless-steel container (5 mL) with a stainless-steel ball (10 mm diameter) and milled in air twice during 90 min at 10 Hz.

K. Graphitization of CNF (gCNF):

100 mg of CNF were placed in an alumina crucible and heated under Ar atmosphere. In a typical experiment, the sample was initially purged for 30 min at room temperature, heated up to 1000 ºC with a 10 ºC/min ramp and hold for 90 min. The sample was then cooled down under Ar atmosphere, yielding 77 mg gCNF.

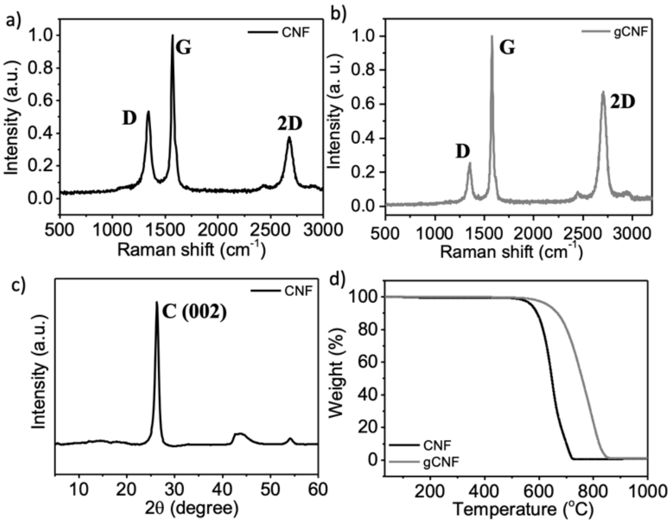

Figure S3. a) Raman spectrum of CNF showing D, G and 2D band at 1353, 1582 and 2714 cm^{-1}, with I_D/I_G of 0.30. **b)** Raman spectrum of gCN showing D, G and 2D band at 1353, 1582 and 2714 cm^{-1}, with I_D/I_G of 0.25. **c)** Powder XRD pattern of CNF showing the graphite planes at 26° (001), 43° (001), 44° (101) and 54° (004). **b)** TGA measurements of CNF and gCNF at a scan rate of 5°C/min in air.

Supplementary Figures

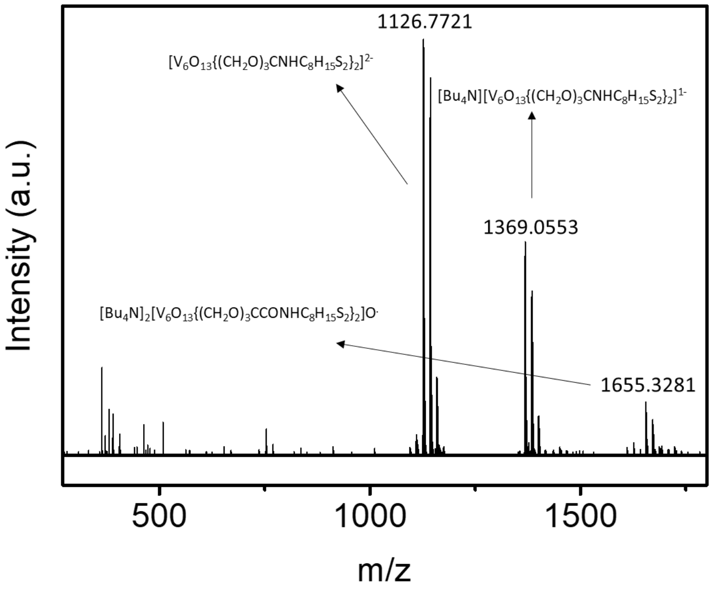

Figure S4. Electrospray ionization (ESI) Mass spectrometry (MS) spectrum for the compound $[Bu_4N]_2[V_6O_{13}\{(CH_2O)_3CNHCOC_8H_{15}S_2\}_2]$ (POM) in positive mode.

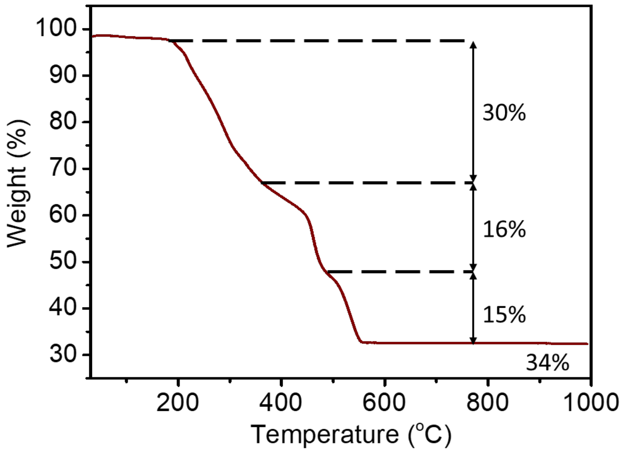

Figure S5. TGA measurements of POM in air (scan rate: 5°C/min). The weight loss between 150-400°C corresponds to the decomposition of $[Bu_4N]^+$ cations and between 400-500°C (two-step process) to the loss of two $(CH_2O)_3CNHCOC_8H_{15}S_2$ fragments. The final residue at 1000 °C is 34% that correspond to vanadium oxide.

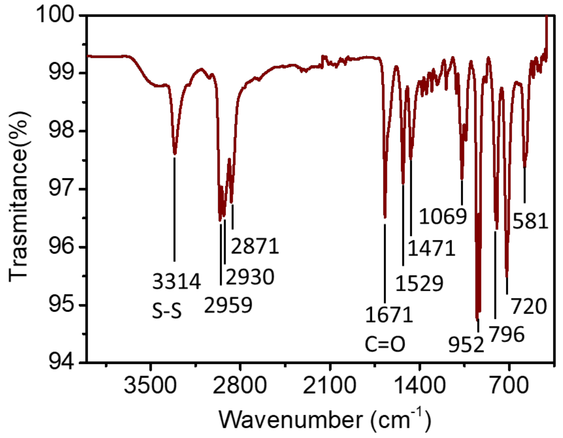

Figure S6. FT-IR measurements of the POM. Peak assignment: 3314 cm^{-1} (S-S bond), 2950-2871 cm^{-1} (C-H bonds), 1671cm^{-1} (C=O bond), 1529-1471 cm^{-1} (C-C bonds), 1069-1040 cm^{-1} (C-O bonds), 952-932 cm^{-1} (V=O bonds), 769, 720 and 581 cm^{-1} (V-O-V bridges).

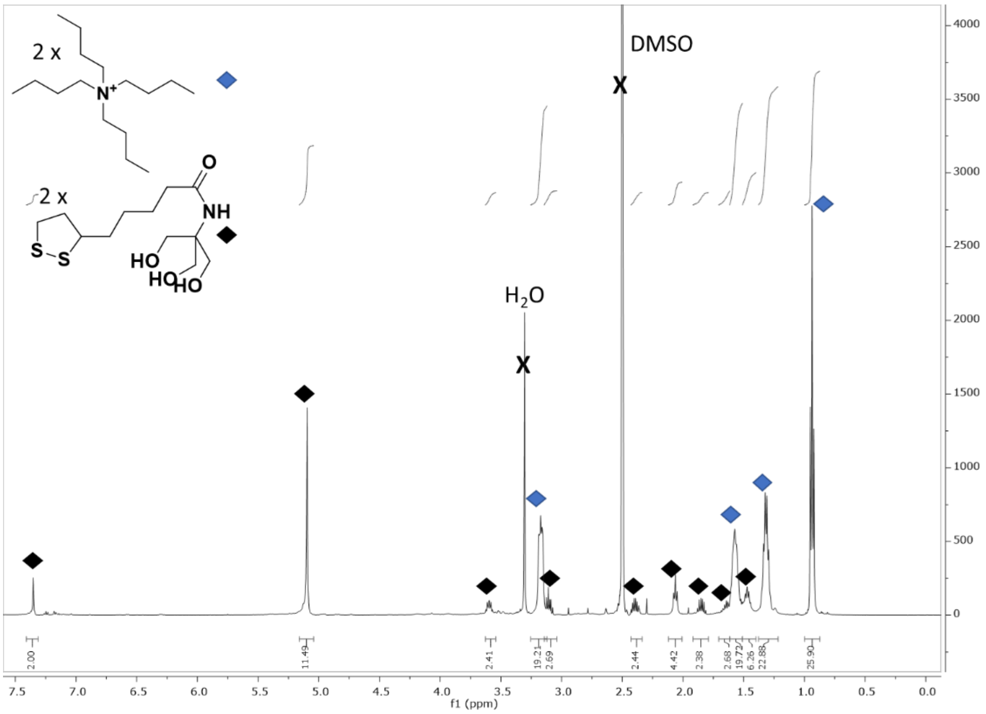

Figure S7. ^{1}H NMR measurements of the POM in DMSO-d_6 at 300 MHz.

Table S1. The crystallographic data obtained by single-crystal X-ray diffraction and structure refinement for POM.

	**POM**
**Chemical Formula**	$C_{56}H_{112}N_4O_{21}S_4V_6$
**Size (mm^3)**	0.027 x 0.042 x 0.159
**Formula weight**	1611.36
**Crystal system**	Monoclinic
**Space group**	$P2_1/n$
**a [Å]**	10.6356(5)
**b [Å]**	19.0646(8)
**c [Å]**	19.9100(9)
**α [°]**	90
**β [°]**	104.612(2)
**γ [°]**	90
**V [Å^3]**	3906.4(3)
**Z**	2
**Theoretical density [mg/m^3]**	1.370
**Temperature [K]**	100
**Tmax/Tmin**	0.9770, 0.8750
**Absorption coeff. [mm^{-1}]**	0.860
**F (000)**	1692
**Θ range [°]**	2.25 to 28.28
**Reflections collected**	68214
**Independent reflections**	9677
**Goodness-of-fit on F2**	1.027
**Final R indices [I > 2σ(I)]**	R1 = 0.0655, Wr2 = 0.1276
***R* indices (all data)**	R1 = 0.1189, Wr2 = 0.1468

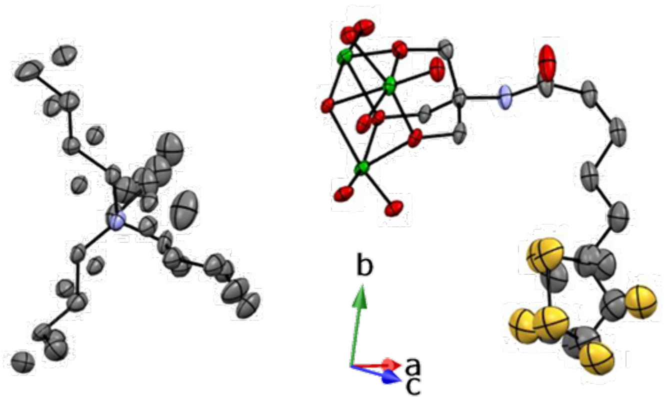

Figure S8. Oak Ridge Thermal Ellipsoid Plot (ORTEP) representation (50% of probability) of the asymmetric unit of POM. Hydrogen atoms are omitted for clarity. Green, blue, yellow, grey and red spheres are depicted for vanadium, nitrogen, sulphur, carbon and oxygen atoms.

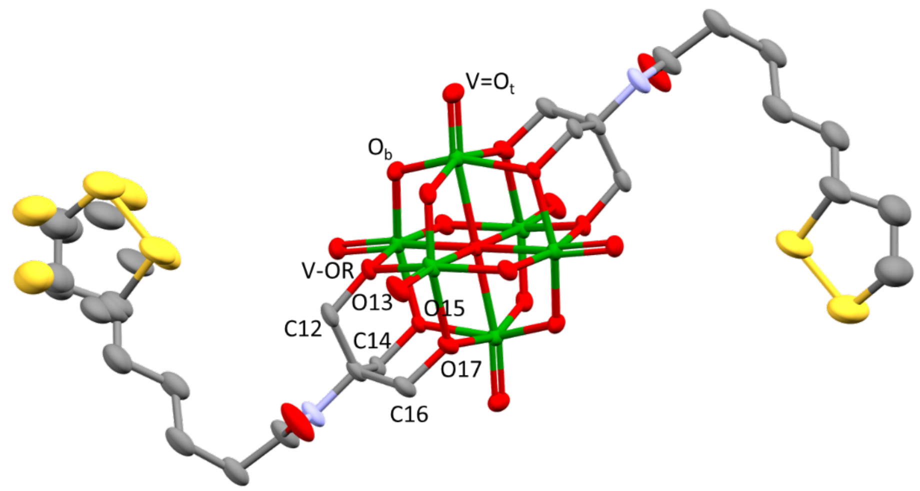

Figure S9. ORTEP representation (50% of probability) of the sulphur-functionalized POM cluster. Hydrogen atoms are omitted for clarity. Green, blue, yellow, grey and red spheres are depicted for vanadium, nitrogen, sulphur, carbon and oxygen atoms.

Table S2. V-O lengths in POM.[3]

V-O bond	Lengths (Å)
$V{=}O_t$	1.598(3)-1.614(3)
$V{-}O_b$	1.746(3)-2.2452(6)
V-OR	1.955(3)-2.067(3)

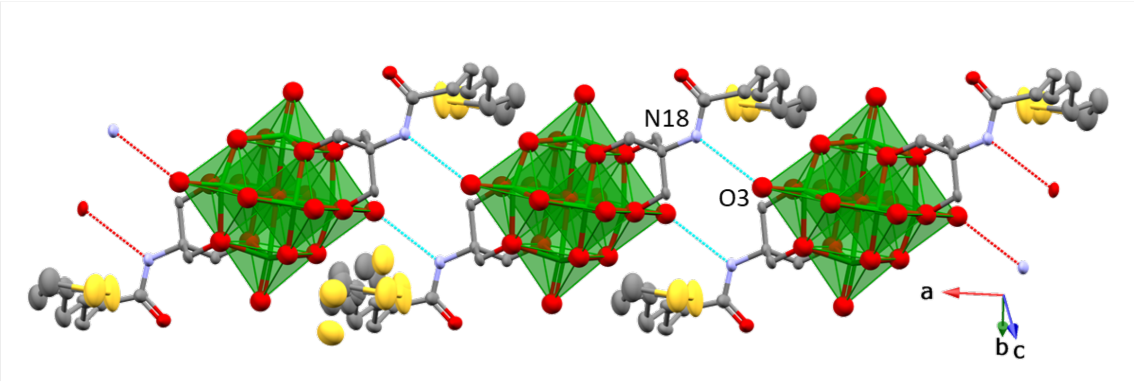

Figure S10. 2D chains of POM clusters are formed through hydrogen bonding interactions between O3···H18-N18 (O3···H18 = 2.25 (5) Å; N18-H18 = 0.80(5) Å) along the *a* axis. Tetrabutylammonium cations and hydrogen atoms are omitted for clarity. Green, blue, yellow, grey and red spheres are depicted for vanadium, nitrogen, sulphur, carbon and oxygen atoms.

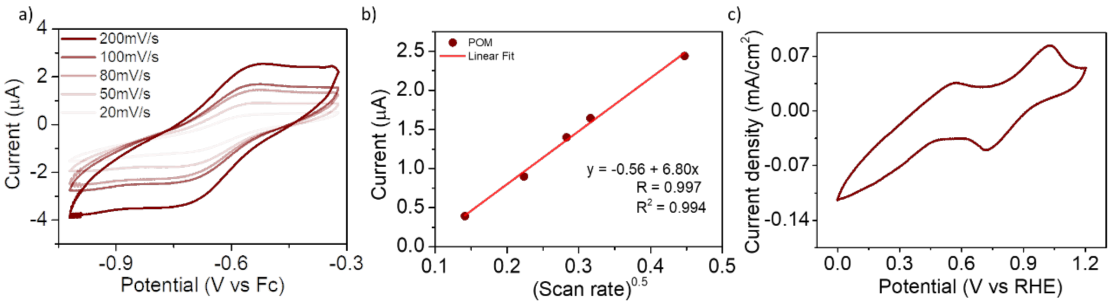

Figure S11. a-b) CV of a POM solution (1 mM) in acetonitrile under nitrogen at different scan rates, indicating a mass-transport limited redox process. Diffusion coefficient of 1.3×10^{-7} cm^2/s was determined using the Randles-Sevick relationship. **c)** CV of POM deposited on a glassy carbon electrode under nitrogen atmosphere in 1 M KOH at 100 mV/s showing the V^V/V^{IV} pair redox. [4]

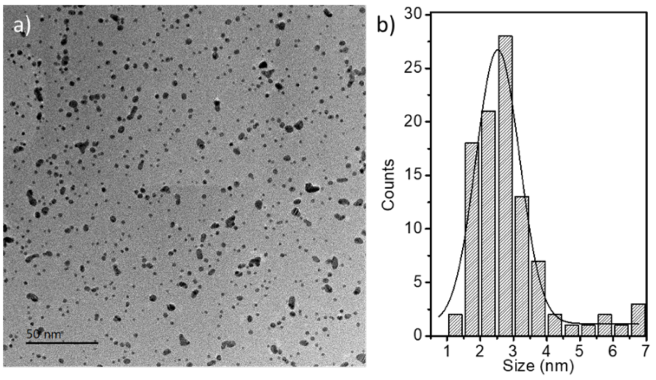

Figure S12. a) HR-TEM of AuNP@C_8S at 80 kV using a copper grid and **b)** particle size distribution histogram of nanoparticles in AuNP@C_8S, showing an average size of 2.5 ± 1.3 nm.

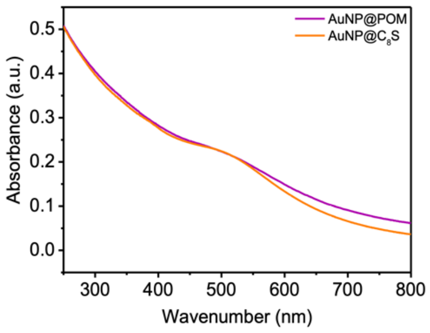

Figure S13. UV-Vis measurements of AuNP@C_8S and AuNP@POM in hexane at 25ºC. The position and size of the band indicates only small variations on the particle size.

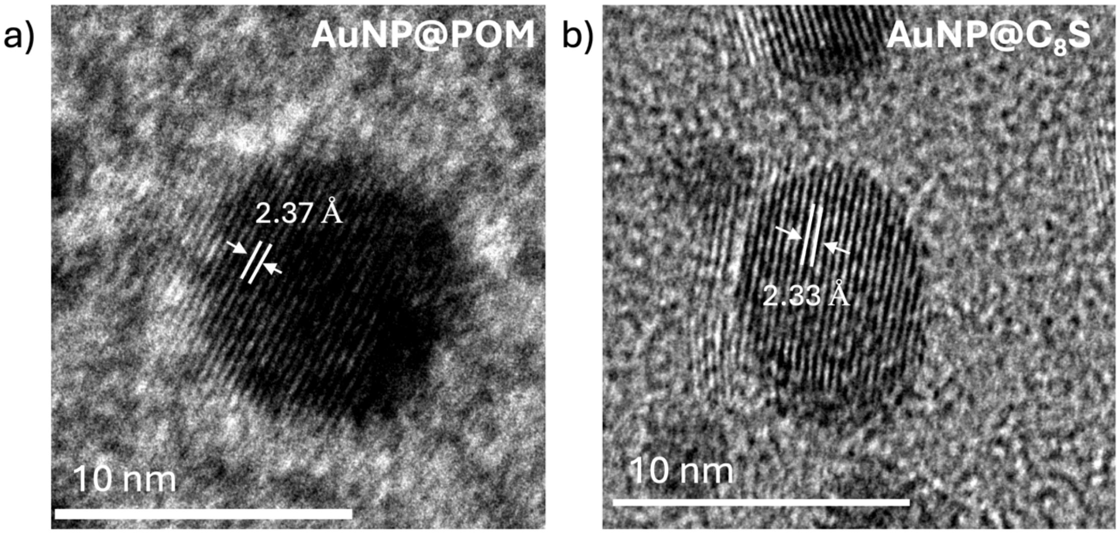

Figure S14. HR-TEM images at 80 kV in a copper grid of **a)** AuNP@POM and **b)** AuNP@C_8S.

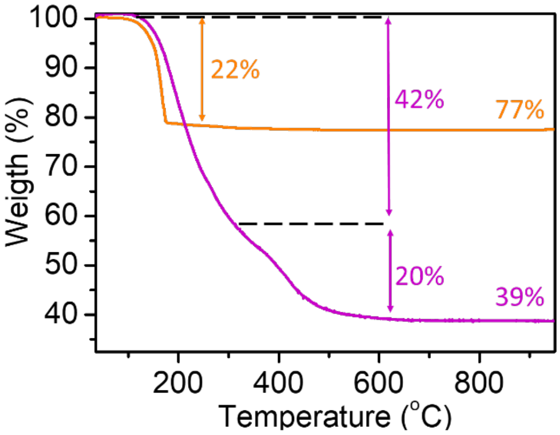

Figure S15. Comparison of the TGA measurements for AuNP@POM and AuNP@C_8S in air (scan rate: 5°C/min). While only one weight loss between 100-200°C, assigned to the $C_8H_{18}S$ surfactant loss, is observed for AuNP@C_8S, two weight losses are observed for AuNP@POM (between 120-342°C assigned to the decomposition of $C_8H_{18}S$ and one $(CH_2O)_3CNHCOC_8H_{15}S_2$, and between 343-561°C corresponding to the loss of the second $(CH_2O)_3CNHCOC_8H_{15}S_2$ fragment). The final residue at 1000°C is 39% and 77% for AuNP@POM and AuNP@C_8S, respectively.

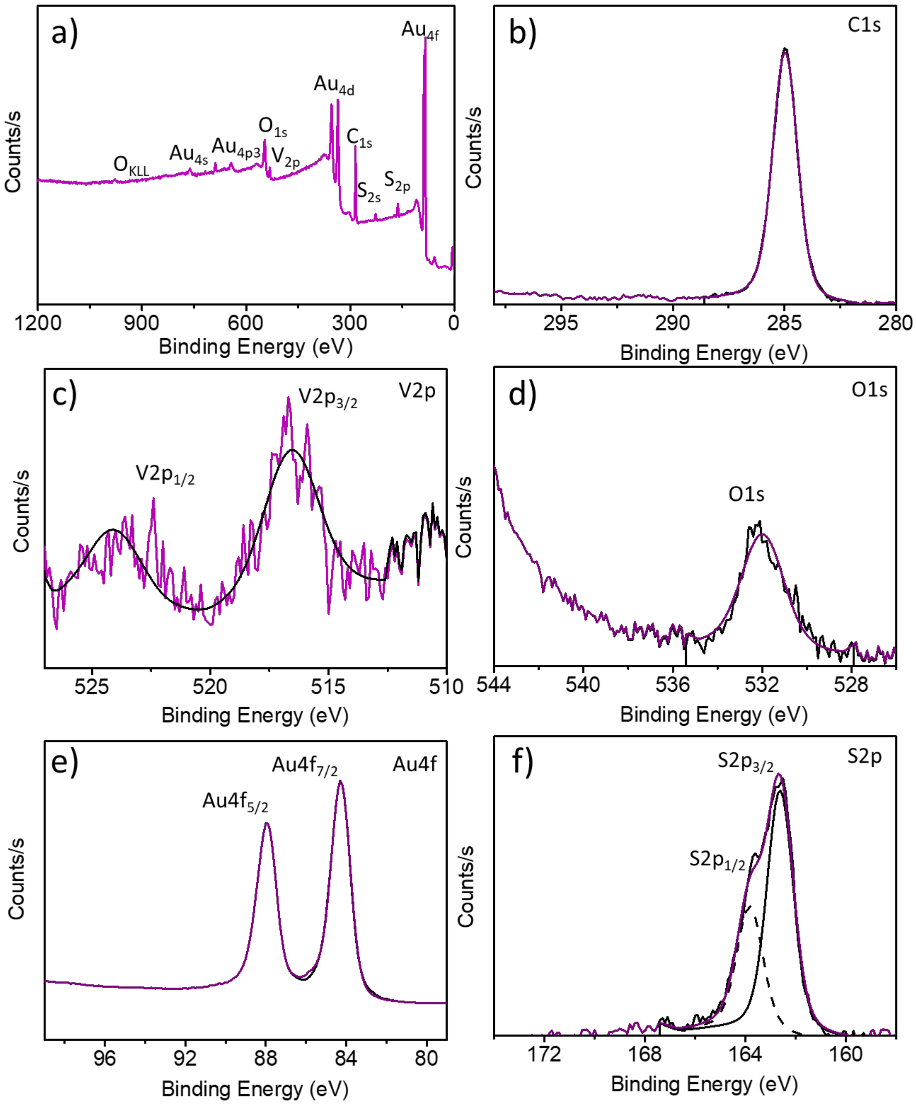

Figure S16. a) XPS spectra wide range spectrum of AuNP@POM showing 69.72% C1s, 5.43% O1s, 6.5% $S2p_{3/2}$, 14.73% $Au4f_{7/2}$, and 0.27% $V2p_{3/2}$. Energy regions for C1s (**b**), V2p (**c**) (515 eV confirms the presence of V-O bond and the oxidation state of the V as (V) [6][7]), O1s (**d**), Au4f (**e**) and S2p (**f**) (162.7 eV corresponds to S-Au bond (typical of lipoic acid chains) [8]). The ratios Au/V and Au/S are *ca.* 54/1 and 2.26/1, respectively, with the Au/S ratio being similar to that observed for AuNP@C_8S.

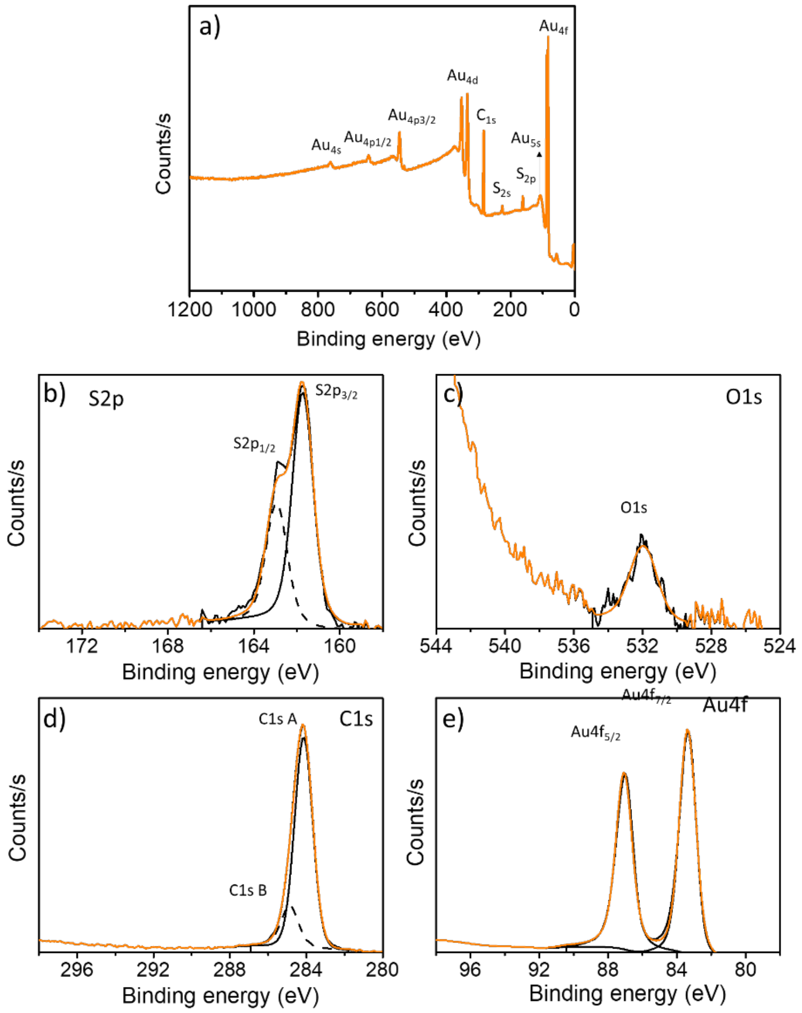

Figure S17. a) XPS spectra wide range spectrum of AuNP@C_8S showing 18.74% Au4$f_{7/2}$, 78.69% C1s 1.56% O1s and 8.02% S2$p_{3/2}$ in atomic percentage. Energy regions for S2p (**b**), O1s (**c**), C1s (**d**) and C1s (**d**) (161.7 eV corresponds to S-Au bond typical of aliphatic chains [5]). The Au/S ratio is 2.

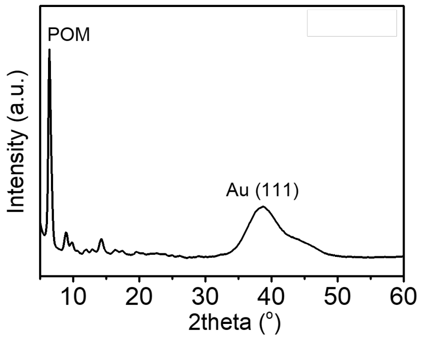

Figure S18. Powder XRD pattern of a physically mixture of POM with preformed octanethiol gold nanoparticles (AuNP@C_8S/POM).

Table S3. Comparison of the ORR activity of all materials at 1600 rpm in an oxygen-saturated 1 M KOH solution.

j_k (mA/cm^2)	n (K-L)	$E_{1/2}$ (V)	η (V)	Tafel ($mV \cdot dec^{-1}$)	E_0 (mV)	j_L (mA/cm^2)	n (0.3V)	HO_2^- % (0.3V)	Material
**0.25**	**1.80**	**0.58**	**0.332**	**-147.9**	**668**	**-0.7**	**2.56**	**25**	**POM**
0.69	2.07	0.64	0.295	-89.3	705	-0.9	3.14	43	AuNP@C_8S
**1.04**	**3.60**	**0.66**	**0.247**	**-168.8**	**753**	**-0.9**	**3.77**	**11**	**AuNP@POM**
1.38	2.07	0.62	0.245	-177.6	746	-1.7	2.47	75	CNF
**1.63**	**3.85**	**0.79**	**0.132**	**-47.4**	**868**	**-1.6**	**3.38**	**30**	**AuNP@POM/CNF**
2.22	4.60	0.68	0.130	-50.7	820	-1.3	3.00	14	AuNP@POM/gCNF
0.34	2.70	0.64	0.269	-103.2	731	-0.8	3.49	30	AuNP@C_8S/CNF
0.85	4.10	0.86	0.066	-82.8	934	-2.1	3.73	13	Pt/C

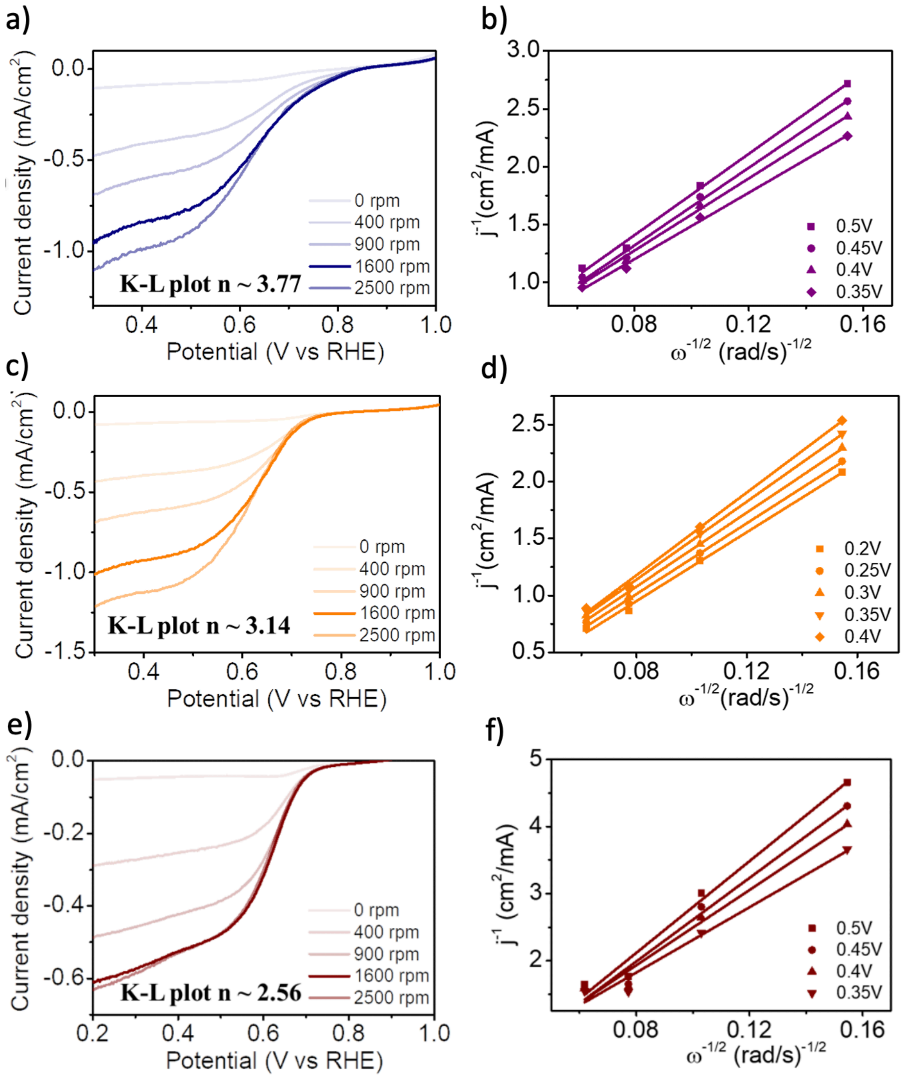

Figure S19. LSV measurements at different rotating speeds (0-2500 rpm) in an oxygen-saturated aqueous electrolyte (1M KOH) and Koutecky-Levich plots for AuNP@POM **(a-b)**, AuNP@C_8S **(c-d)** and POM **(e-f)**, respectively. The number of transferred electrons (*n*) and the kinetic current density (j_k), calculated using the K-L equation (eq. 2), are 2.07 and 1.04 mA/cm^2 for AuNP@POM; 2.70 and 0.69 mA/cm^2 for AuNP@C_8S and 1.80 and 0.25 mA/cm^2 for POM.

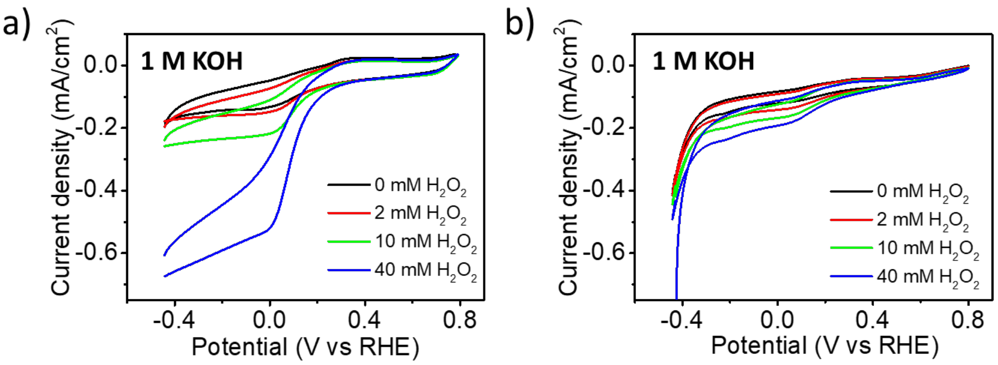

Figure S20. CV measurements of POM (**a**) and AuNP@C_8S (**b**) at different concentrations of H_2O_2 in 1 M KOH with a scan rate of 50 mV/s, confirming the higher electrocatalytic activity of POM towards the hydrogen peroxide reduction compared to AuNP@C_8S.

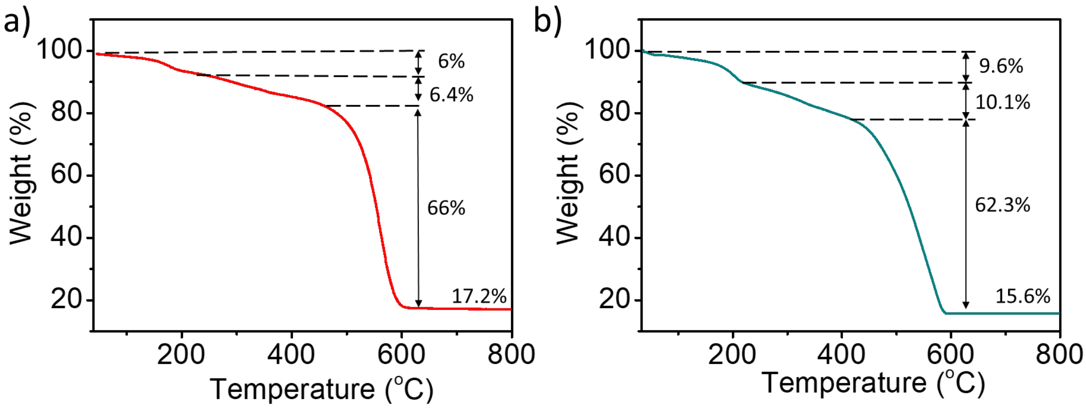

Figure S21. Comparison of the TGA measurements of AuNP@POM/CNF **(a)** with AuNP@POM/gCNF **(b)** in air (scan rate: 5 ºC/min). The weight loss for both materials occurs in three steps: between 100 and 220 °C, attributed to the loss of surfactant ($C_8H_{18}S$), between 220 and 410 °C, corresponding to the loss of two $(CH_2O)_3CNHCOC_8H_{15}S_2$ fragments, and between 410 and 600 °C attributed to the oxidation of CNF. At 800 °C, the final residue is 17.2% for AuNP@POM/CNF and 15.6.% for AuNP@POM/gCNF.

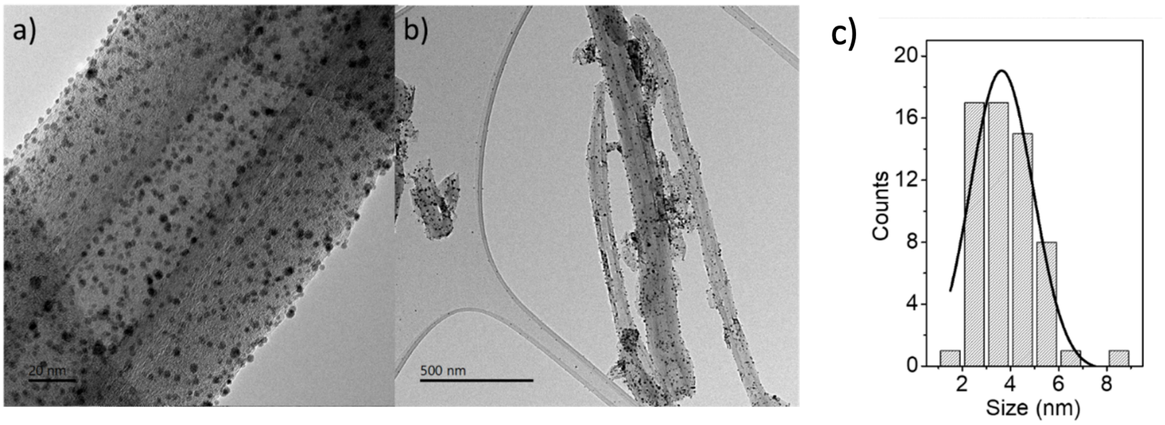

Figure S22. a-b) HR-TEM images at 80 kV of AuNP@POM/CNF using a copper grid. **c)** Particle size distribution histogram of AuNP@POM on AuNP@POM/CNF, showing an average size of 3.6 ± 2.6 nm.

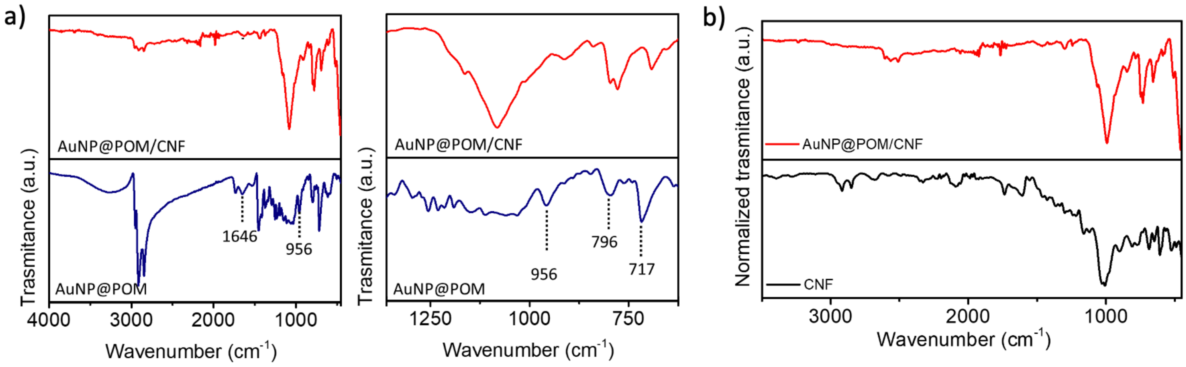

Figure S23. FT-IR spectrum of AuNP@POM/CNF compared with AuNP@POM and CNF.

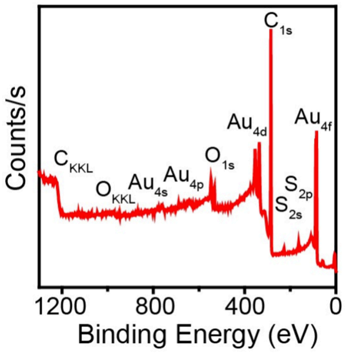

Figure S24. A wide range region for the XPS measurements of AuNP@POM/CNF

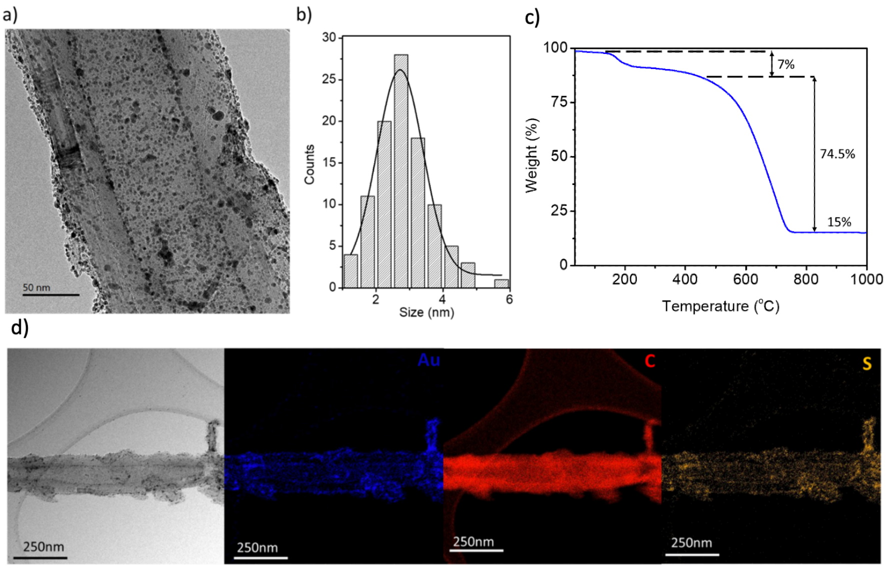

Figure S25. a) HR-TEM at 80 kV of AuNP@C_8S/CNF in a copper grid. **b)** Particle size distribution histogram of the AuNP@C_8S nanoparticles in AuNP@C_8S/CNF with an average particle size of 2.7 ± 1.7 nm. **c)** TGA measurements of AuNP@C_8S/CNF in air at 5 °C/min. The weight lost between 105-270 °C corresponds to the loss of surfactant (C_8H_{18}SH) and between 387-783 °C to the oxidation of CNF, giving a final residue of 15% of Au. **d)** STEM/EDS elemental mapping of Au, C and S for AuNP@C_8S/CNF.

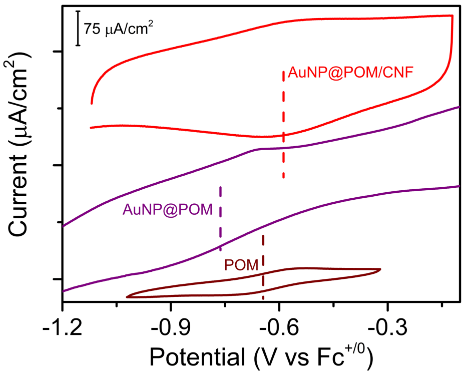

Figure S26. CV for AuNP@POM/CNF and AuNP@POM on a glassy carbon electrode in acetonitrile show a one-electron process associated to the V^{V}/V^{IV} redox couple [4] with a formal potential (vs Ferrocene) of -0.545 V (peak separation of 144 mV) and -0.759 V (peak separation of 203 mV), respectively. For comparison, CV of 1 mM POM in acetonitrile solution shows similar redox process with a formal potential of -0.650 V (vs Ferrocene) and peak separation of 214 mV. [4] The shift to more negative potential of the formal potential of AuNP@POM with respect to POM is in agreement with the electron transfer from the Au surface to the POM inferred from IR and XPS measurements.

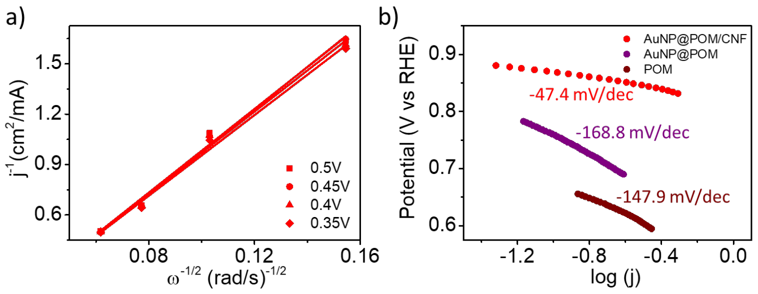

Figure S27. a) Koutecký-Levich (K-L) plot for AuNP@POM/CNF. The number of transferred electrons (*n*) and the kinetic current density (j_k), calculated using the K-L equation (eq. 2), are 3.85 and 1.63 mA/cm^2, respectively. **b)** Tafel slopes of AuNP@POM/CNF, AuNP@POM and POM.

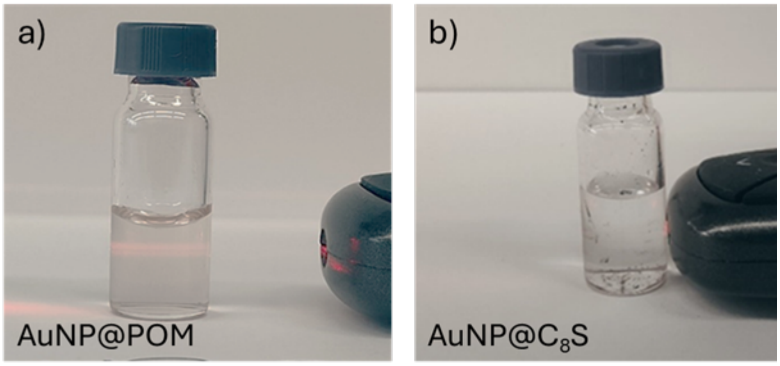

Figure S28. The zeta potential of AuNP@POM in water (0.1 mg/mL) (a) was found to be significantly higher in absolute value (-55.2 ± 2.0 mV) compared to AuNP@C_8S under the same conditions (b) (-3.62 ± 1.06 mV), confirming the higher hydrophilic nature for the POM-functionalized nanoparticles. The increased hydrophilicity of AuNP@POM in water was also evidenced by the formation of colloidal dispersions in water (Tyndall effect).

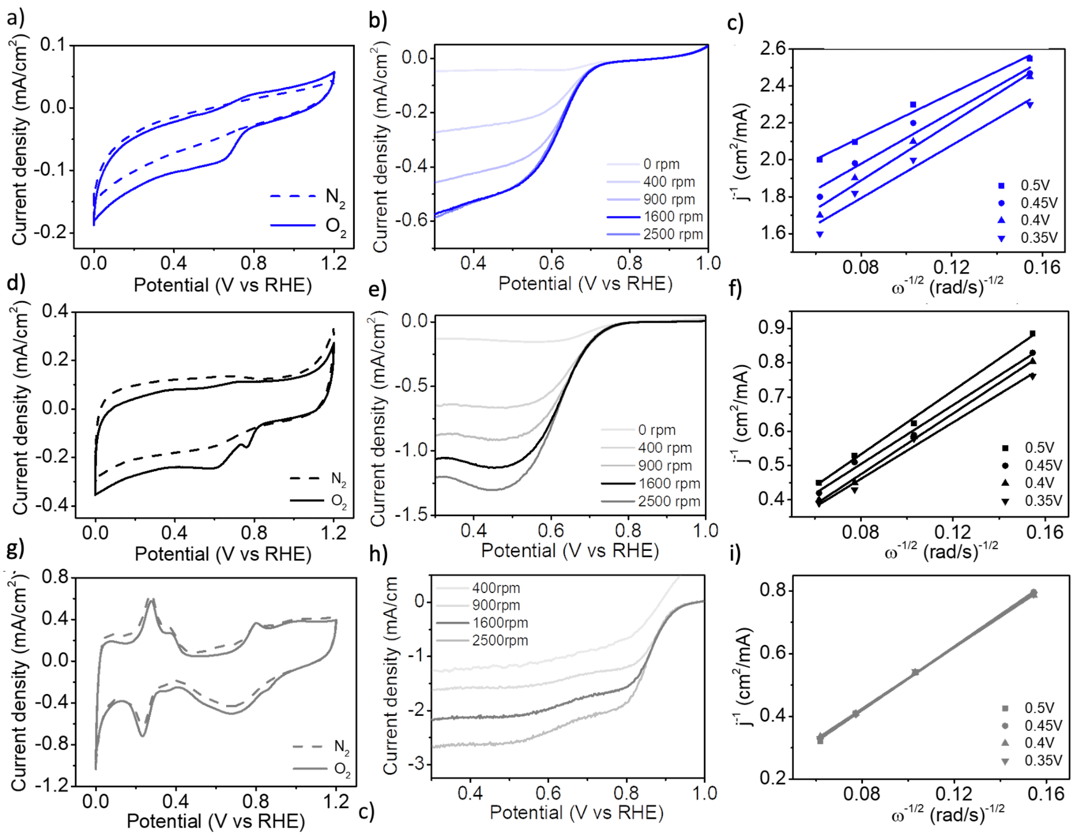

Figure S29. CV in both an oxygen- and nitrogen-saturated electrolyte (1M KOH) (left), LSV at different rotating speeds (0-2500 rpm) in an oxygen-saturated electrolyte (1M KOH) (middle) and K-L plots (right) for AuNP@C_8S/CNF **(a-c)**, CNF (**d-f**) and Pt/C **(g-i)** deposited in a glassy carbon electrode. The number of transferred electrons (*n*) and the kinetic current density (j_k), calculated using the K-L equation (eq. 2), are 2.70 and 0.34 mA/cm^2; 2.07 and 1.38 mA/cm^2; 4.10 and 0.85 mA/cm^2 for AuNP@C_8S/CNF, CNF and Pt/C, respectively.

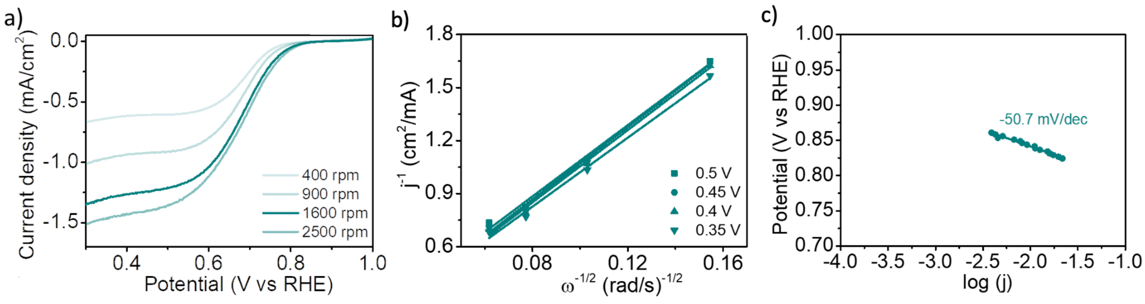

Figure S30. a) LSV curves of AuNP@POM/gCNF in an oxygen-saturated electrolyte (1M KOH) at different rotating rates with the corresponding **b)** K-L and **c)** Tafel plot. The number of transferred electrons (*n*) and the kinetic current density (j_k), calculated using the K-L equation (eq. 2), are 4.60 and 2.22 mA/cm^2, respectively.

Table S4. Stability comparison of AuNP@POM/CNF with different electrocatalyst for ORR reported in the last two years.

Sample	Electrolyte	Retention (%)	Duration (h)	Reference
**POM@AuNPs/CNF**	**1M KOH**	**80**	**24.0**	**This study**
**Pt/C**	**1M KOH**	**16**	**24.0**	**This study**
Co4POM@FeOOH-P	1M KOH	93	50.0	[9]
SiW12@FeOOH-P	1M KOH	50	50.0	[9]
MnSe@MWNCT	1M KOH	95.04	12.0	[10]
N-GRW	1M KOH	90	12.0	[11]
MnPc/FCNT800	1M KOH	87.9	20	[12]

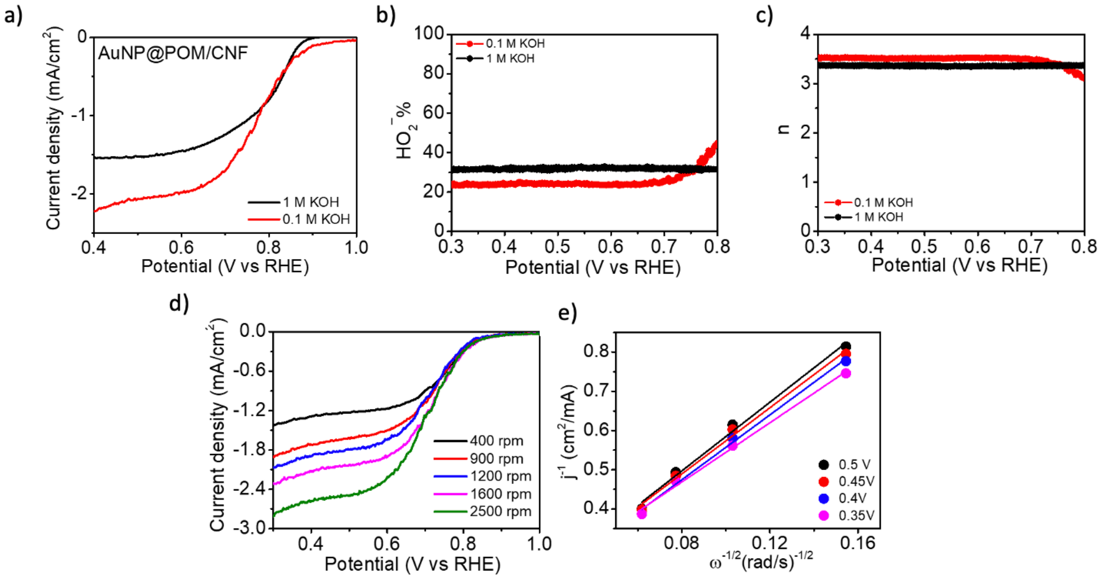

Figure S31. a-c) LSV curves, the variation of the HO_2^-% and the number of electrons (*n*) at 1600 rpm for AuNP@POM/CNF at a scan rate of 10 mV/s in an oxygen-saturated 1M and 0.1 M KOH solution. The values of n and HO_2^-% are 3.51 and 23.3%, respectively, for 0.1 M KOH and 3.38 and 30%, respectively, for 1 M KOH. **d-e**) LSV curves at different rotating speeds for AuNP@POM/CNF at a scan rate 10 mV/s in an oxygen-saturated 0.1 M KOH solution and the corresponding K-L plots.

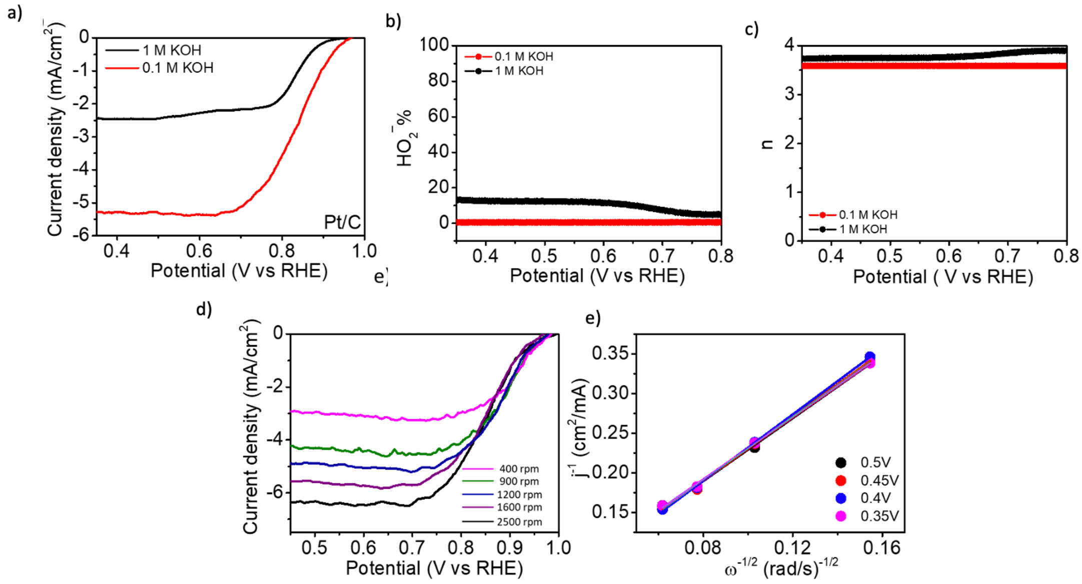

Figure S32. a-c) LSV curves, the variation of the HO_2^-% and the number of electrons (*n*) at 1600 rpm for Pt/C at a scan rate of 10 mV/s in an oxygen-saturated 1M and 0.1 M KOH solution. The values of n and HO_2^-% are 3.52 and 1%, respectively for 0.1 M KOH and 3.73 and 13%, respectively, for 1 M KOH. **d-e**) LSV curves at different rotating speeds for Pt/C at a scan rate 10 mV/s in an oxygen-saturated 0.1 M KOH solution and the corresponding K-L plots (n and j_k values are 3.58 and 4.54 mA/cm^2 respectively.).

References.

[1] H. S. Rho, H. S. Baek, D. H. Kim, and I. S. Chang, Bull. *Korean. Chem. Soc.*, **2006**, *27*, 584-586.

[2] Kubas, G. J.; Hoff, C.; Hlatky, G. G.; Crabtree, R. H.; Kubat-Martin, K. A.; Kubas, G.; Cameron, C.; Moehring, G.; Walton, R.; Caulton, K., Inorganic Syntheses, Volume 27.

[3] M. Schidler, F. C. Hawthorne, and W. H. Baur, *Chemistry of Materials*, **2000**, *12*, 1248-1259.

[4] Salazar Marcano, D.E.; Kalandia, G; Aly Moussawi, M; Van Hecke, K.; Parac-Vogt, T.N., *Chem. Sci*., **2023**, *14*, 5405-5414

[5] Z. Zhuang, Y. Zhang, L. Hu, H. Ying, and W. Han, *Chem. Asian J*, **2020**, *15*, 2153-2159.

[6] R. Manikadan, C. J. Raj, M. Rajesh, B. C. Kim, J. Y. Sim, and K. H. Yu, *ChemElectroChem*, **2018**, *5*, 101-111.

[7] H-H. Liu, H-L. Zhang, H-B. Xu, T-P. Lou, Z. T. Sui, and Y. Zhang, *Ceramics International*, **2018**, *44*, 1583-1588.

[8] A. A. Volkert, V. Subramanian, M. R. Ivanov, A. M. Goodman, and A. J. Haes, *ACS Nano*, **2011**, *5*, 4570-4580.

[9] W. Shang, Y. Wang, Y. Jiang, M. Wu, M. Zeng, P. Wang, L. Qiu, Z. Jia, *Applied Catalysis A: General* **2022**, *644*, 118810.

[10] H. Singh, M. Marley-Hines, S. Chakravarty, M. Nath, *Journal of Materials Chemistry A* **2022**, *10*, 6772-6784.

[11] H. B. Yang, J. Miao, S.-F. Hung, J. Chen, H. B. Tao, X. Wang, L. Zhang, R. Chen, J. Gao, H. M. Chen, *Science advances* **2016**, *2*, e1501122.

[12] Z. Zheng, X. Hong, D. Wu, N. Sun, Y. Kuang, D. Zhang, X. Yao, P. Du, K. Huang, M. Lei, *Advanced Composites and Hybrid Materials* **2023**, *6*, 1-13.
